# Genomics of *Staphylococcus aureus* ocular isolates

**DOI:** 10.1371/journal.pone.0250975

**Published:** 2021-05-03

**Authors:** William L. Johnson, Michael B. Sohn, Samantha Taffner, Payel Chatterjee, Paul M. Dunman, Nicole Pecora, Rachel A. F. Wozniak

**Affiliations:** 1 Department of Ophthalmology, University of Rochester School of Medicine and Dentistry, Rochester, New York, United States of America; 2 Department of Biostatistics and Computational Biology, University of Rochester School of Medicine and Dentistry, Rochester, New York, United States of America; 3 Department of Clinical Microbiology, University of Rochester School of Medicine and Dentistry, Rochester, New York, United States of America; 4 Department of Microbiology and Immunology, University of Rochester School of Medicine and Dentistry, Rochester, New York, United States of America; Universidade de Lisboa Faculdade de Medicina, PORTUGAL

## Abstract

*Staphylococcus aureus* is a major cause of ocular infections, often resulting in devastating vision loss. Despite the significant morbidity associated with these infections, little is yet known regarding the specific strain types that may have a predilection for ocular tissues nor the set of virulence factors that drive its pathogenicity in this specific biological niche. Whole genome sequencing (WGS) can provide valuable insight in this regard by providing a prospective, comprehensive assessment of the strain types and virulence factors driving disease among specific subsets of clinical isolates. As such, a set of 163-member *S*. *aureus* ocular clinical strains were sequenced and assessed for both common strain types (multilocus sequence type (MLST), *spa*, *agr*) associated with ocular infections as well as the presence/absence of 235 known virulence factors in a high throughput manner. This ocular strain set was then directly compared to a fully sequenced 116-member non-ocular *S*. *aureus* strain set curated from NCBI in order to identify key differences between ocular and non-ocular *S*. *aureus* isolates. The most common sequence types found among ocular *S*. *aureus* isolates were ST5, ST8 and ST30, generally reflecting circulating non-ocular pathogenic *S*. *aureus* strains. However, importantly, ocular isolates were found to be significantly enriched for a set of enterotoxins, suggesting a potential role for this class of virulence factors in promoting ocular disease. Further genomic analysis revealed that these enterotoxins are located on mobile pathogenicity islands, thus horizontal gene transfer may promote the acquisition of enterotoxins, potentially amplifying *S*. *aureus* virulence in ocular tissues.

## Introduction

Bacterial ocular infections such as conjunctivitis, keratitis (corneal infection), or endophthalmitis are a major cause of ophthalmic morbidity and vision loss, collectively contributing to over 5 million health care visits and nearly 1 billion dollars in associated health care costs [1–6]. *Staphylococcus aureus*, a major human pathogen, is responsible for nearly 70% of ocular and peri-orbital infections [7–12], often resulting in rapid, permanent, ocular tissue damage. Much is still unknown regarding the mechanisms by which *S*. *aureus* can establish and maintain infections in the eye, however, initial studies have begun to identify specific strain types as well as virulence factors that may be particularly important in ocular disease. For example, multilocus sequence typing (MLST), which compares the genetic sequences of seven housekeeping genes (*arcC*, *aroE*, *glpF*, *gmk*, *pta*, *tpi*, and *yqiL*) to identify sequence types (STs), has revealed ST5, ST8, ST15, ST30, ST59, and ST772 as common among specific *S*. *aureus* ocular strain sets [13–16]. Additionally, studies to assess the prevalence of *S*. *aureus* virulence factors among ocular isolates such as Panton-Valentine Leukocidin (*pvl*), Enterotoxin E (*sea*) or Leukocidin E (*lukE*) have demonstrated that while *pvl* and *lukE* are found in the majority of sampled ocular strains, *sea* may be less common [16, 17]. While this initial work has provided insight into the genetic characteristics of ocular *S*. *aureus* clinical isolates, they have largely been limited to small sample sizes and a handful of virulence factors. Given the high prevalence and associated morbidity of *S*. *aureus* ocular infections, it is critical to broaden our understanding of the specific lineages as well as the genetic determinants associated with ocular infection through the analysis of a large, diverse, contemporary clinical isolate strain set. Doing so will lead to significant advances in understanding *S*. *aureus* pathogenesis, improve disease surveillance as well as provide opportunities for the development of novel therapeutics to treat these blinding infections.

A genomics-based approach can be particularly advantageous in this regard by providing a non-biased, empiric approach to identifying genetic similarities and differences among clinical isolates. In particular, bacterial whole genome sequencing (WGS) is now widely accepted as a the highest-resolution approach for infection control through defining emerging bacterial lineages and population structures, antibiotic resistance determinants, and the genetic basis of virulence [18–20]. Subsequent comparative genomics and genome wide association studies can leverage WGS data from clinical isolates to reveal those lineages and genetic determinants that may be important in the setting of specific infections. For example, WGS has been used successfully to examine *S*. *aureus* isolates collected from the bloodstream, airways, endocarditis and joint infections in order to further understand specific population structures as well as explore the relationship between virulence factors and patient outcomes [21–24]. In the current study, WGS is used to analyze a contemporary set of 163 ocular *S*. *aureus* isolates collected from around the world. A custom analytical pipeline determined MLST, *spa* and *agr* typing in order to further define circulating *S*. *aureus* ocular lineages, as well as the presence or absence of 235 known *S*. *aureus* virulence factors. Ocular isolates were then subsequently compared to a comparably diverse 116-member non-ocular strain set in order to prospectively define key virulence factors that may be important in ocular infections.

## Materials and methods

### Bacterial strains and growth conditions

163 clinical *S*. *aureus* ocular isolates were obtained from the University of Rochester Flaum Eye Institute (n = 14) or commercially from International Health Management Associates (IHMA), Schaumburg, IL (n = 149) (Table 1, S1 Table). For comparison purposes, 116 fully sequenced *S*. *aureus* strains from non-ocular infections with published assembled and annotated whole genome sequences were downloaded from the National Center for Biotechnology Information (NCBI) database (Table 1, S2 Table). Cultures for genomic extraction were grown by inoculating a single colony in Muller Hinton broth (MH) and incubated at 37°C with shaking at 200 rpm.

**Table 1:** Strain characteristics of a 163-member ocular and 116-member non-ocular *S*. *aureus* isolate collection.

Characteristic	*Ocular n (%)*	*Non-Ocular n (%)*
**Gender**		
*Male*	75 (46)	15 (13)
*Female*	79 (49)	4 (3)
*Unknown*	9 (5)	97 (84)
**Age**		
*0–19*	47 (29)	4 (3)
*20–39*	15 (9)	8 (7)
*40–59*	31 (19)	6 (5)
*60–79*	47 (29)	10 (9)
*80–100*	23 (14)	5 (4)
*Unknown*	-	83 (72)
**Source**		
*Cornea*	74 (45)	-
*Conjunctiva*	26 (16)	-
*Eye*	63 (39)	-
*Skin/Soft Tissue*	-	31 (27)
*Blood*	-	22 (19)
*Nasal*	-	14 (12)
*Joint*	-	9 (8)
*Lung*	-	8 (7)
*Perineum*	-	6 (5)
*Bone*	-	4 (3)
*Heart*	-	1 (1)
*Fecal Sample*	-	1 (1)
*Neonatal*	-	1 (1)
*Laboratory Strain*	-	2 (2)
*Non-human*	-	4 (3)
*Unknown*	-	13 (11)
**Geography**		
*North America*	110 (67)	25 (21)
*Europe*	30 (18)	28 (24)
*South America*	9 (6)	30 (26)
*Asia*	3 (2)	30 (26)
*Africa*	2 (1)	1 (1)
*Middle East*	9 (6)	-
*Unknown*	-	2 (2)
**MSSA**	113 (69)	32 (28)
**MRSA**	50 (31)	84 (72)

### Whole genome sequencing

Chromosomal DNA was extracted using the Qiagen DNeasy Blood and Tissue kit (Qiagen, Germantown, MD) following the manufacturer’s protocol, and the resulting genomic DNA was sent to Novogene, Inc. (Davis, CA) for whole genome sequencing utilizing the Illumina NovaSeq 6000 platform (Illumina, Inc., San Diego, CA).

### Analysis of whole genome sequencing data

Raw sequencing reads were processed through a custom analytical pipeline developed at the University of Rochester (S1 Fig). The genus and species of each isolate was confirmed using StrainSeeker version 1.5 [25], while BLASTn version 2.7.1+ [26] was used to perform *in silico* multilocus sequence typing (MLST) [27] via comparison to databases from the Center for Genomic Epidemiology. s*pa* typing for each ocular isolate was conducted using spaTyper (version 1.0) from the Center for Genomic Epidemiology [28], while *agr* typing was conducted using BLAST against representative *agrC* genes from each type (*agr* type I accession: AF210055; *agr* type II accession: AF001782; *agr* type III accession: AF001783; and *agr* type IV accession: AF288215), and confirmed by nucleotide BLAST [29]. SCCmec elements were identified using the SCCmecFinder (version 1.2) tool at the Center for Genomic Epidemiology. Finally, assemblies were compared to a custom virulence factor database consisting of 235 known sequences associated with *S*. *aureus* virulence (S3 Table) using BLASTn version 2.7.1+ [26].

### Single nucleotide polymorphism identification and phylogenetic analysis

Trimmed high quality reads were aligned to the *S*. *aureus* NCTC 8325 reference genome using bowtie2 [30] and transferred to a modified CFSAN SNP Pipeline version 1.0.0. High quality SNPS were identified by the call sites command using SAMtools version 1.5 [31] and combined into a SNP list using the merge_sites function. Areas of unusual SNP density (more than three SNPs in a 50 base pair window) were removed. The resulting SNP list was concatenated and combined into a single multifasta file which was used to generate a maximum likelihood tree using Parsnp version 2.1.10 [32]. The resulting tree was then visualized using the online IToL viewer [33], while MLST data was visualized by goeBURST FullMST using Phyloviz 2.0 [34].

### Enterotoxin gene cluster identification

To identify enterotoxin gene clusters (*egcs*), genomes were annotated using RAST [35, 36] to determine the orientation of constituent genes. *egc1* is readily differentiated by the presence of *Ψent1* and *Ψent2* [37], while *egc2* and *egc3* were differentiated by protein BLAST [38] alignment against *seu* from *S*. *aureus* RF122 (*egc2*, accession number: WP_000764692.1); or *seu*_*v*_ from *S*. *aureus* 383F (*egc3*, accession number: AAP41903.1). *Staphylococcus aureus* pathogenicity islands (SaPIs) were identified using the online Pathogenicity Island Database (PAIDB version 2.0) [39].

### Statistics

Descriptive statistics were used to summarize the characteristics of the strain set and the study population. Means and standard deviations were used for symmetrically distributed continuous data, medians and interquartile ranges for skewed continuous data, and frequencies and percentages for categorical data. The Jaccard distance, which is a commonly used metric for presence/absence data, was used to measure dissimilarity between strains [40], and hierarchical clustering using this distance and Ward’s minimum variance method was performed to group strains together that encoded a similar pattern of virulence factors [41]. To obtain the optimal number of groups or clusters, a 95% bootstrapped confidence interval (CI) of the total within-cluster sum of squares for each number of clusters was constructed and assessed [41, 42]. Fisher’s exact test, Chi-square test, or logistic regression using Firth’s bias reduction method [43] was used for association analysis. Multiple testing correction was performed using the Benjamini-Hochberg (BH) procedure to control the false discovery rate (FDR) [44]. Principal coordinate analysis (PCoA) using the Jaccard distance was used visually to compare dissimilarities of strains [40]. Permutational analysis of variance (PERMANOVA) was used to test dissimilarities between groups of strains (e.g. ocular vs non-ocular) [45]. All analyses were performed with R [46].

## Results

### *S*. *aureus* ocular strain set characteristics

A 163-member *S*. *aureus* ocular isolate strain set was obtained from the University of Rochester Flaum Eye Institute (n = 14) or International Health Management Associates (IHMA) (n = 149) between 2008–2017 (S1 Table). A detailed description of this strain set has been previously published [47], and its core characteristics are presented in Table 1. The majority of isolates were collected from North America (n = 110, 67%), followed by Europe (n = 30, 18%), South America (n = 9, 6%), Middle East (n = 9, 6%), Asia (n = 3, 2%) and Africa (n = 2, 1%). Seventy-five (46%) isolates were collected from male patients, 79 (49%) from female patients and in 9 (6%) cases the gender was unknown. The ages of patients at the time of isolate collection ranged from 0–97, with the majority of patients aged 0–19 (29%) and 60–79 (29%). Seventy-four (45%) isolates were collected from corneal scrapings, 26 (16%) from conjunctival swabs and 63 (39%) were broadly categorized as from eyes, which could include corneal, conjunctival, intracameral and/or intravitreal samples. One hundred and thirteen (69%) were classified as methicillin-sensitive (MSSA) while 50 strains (31%) were classified as methicillin-resistant (MRSA). Multi-drug resistance, as defined by resistance to 3 or more classes of antibiotics, was previously determined to be 24% among the ocular isolates in this study [47].

### Molecular strain typing

#### Multilocus sequence type (MLST)

**Fig 1:**
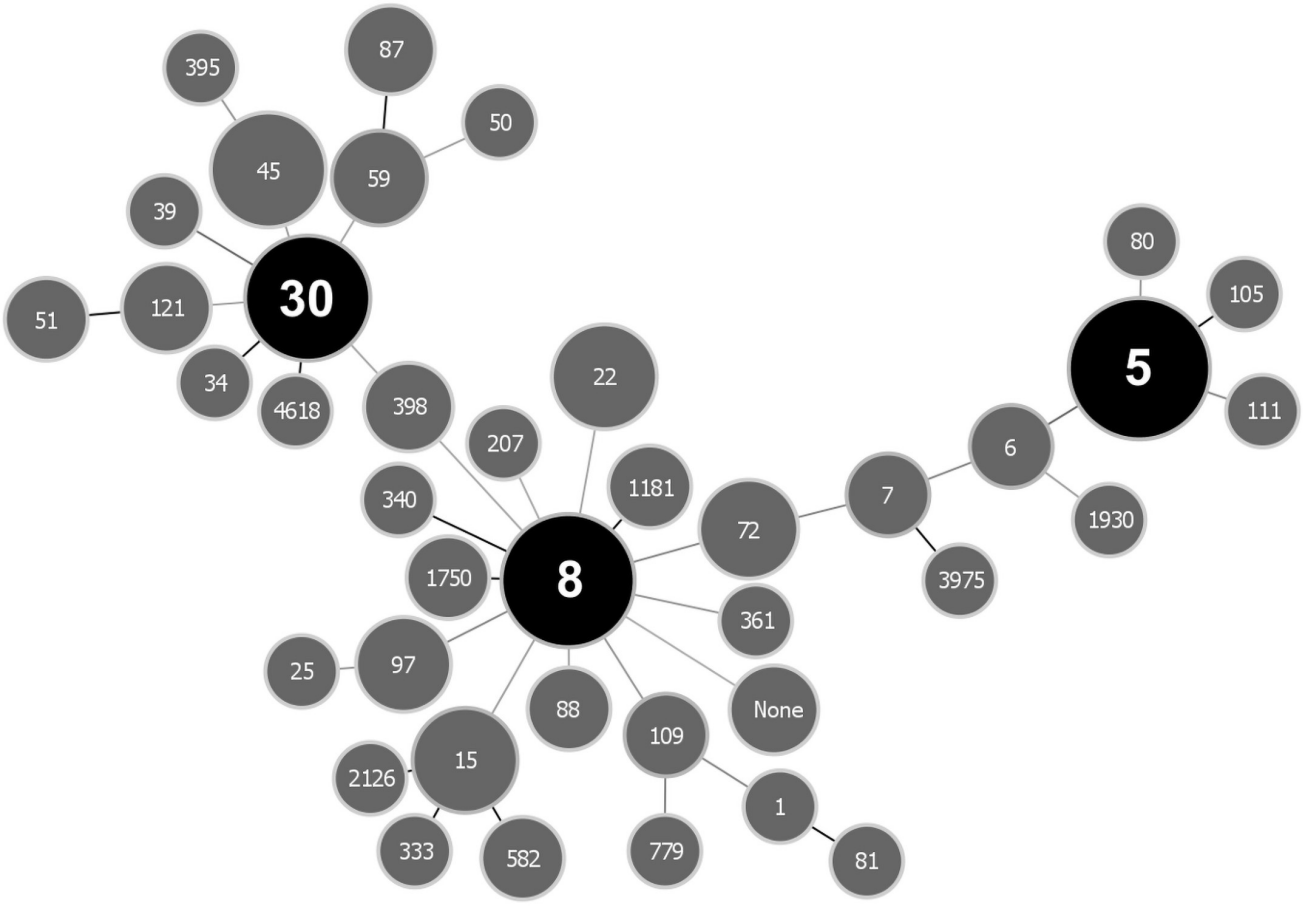
goeBURST full minimum spanning tree of 163 *Staphylococcus aureus* ocular isolate multilocus sequence type (MLST) profiles. The three most abundant MLSTs in each group are shown in black, while all others are shown in grey. Nodes are scaled according to the abundance of each MLST, while connecting links are shown in greyscale to represent the number of MLST allelic differences (ranging from 1 to 7) between strains with darker links representing fewer differences.

**Fig 2:**
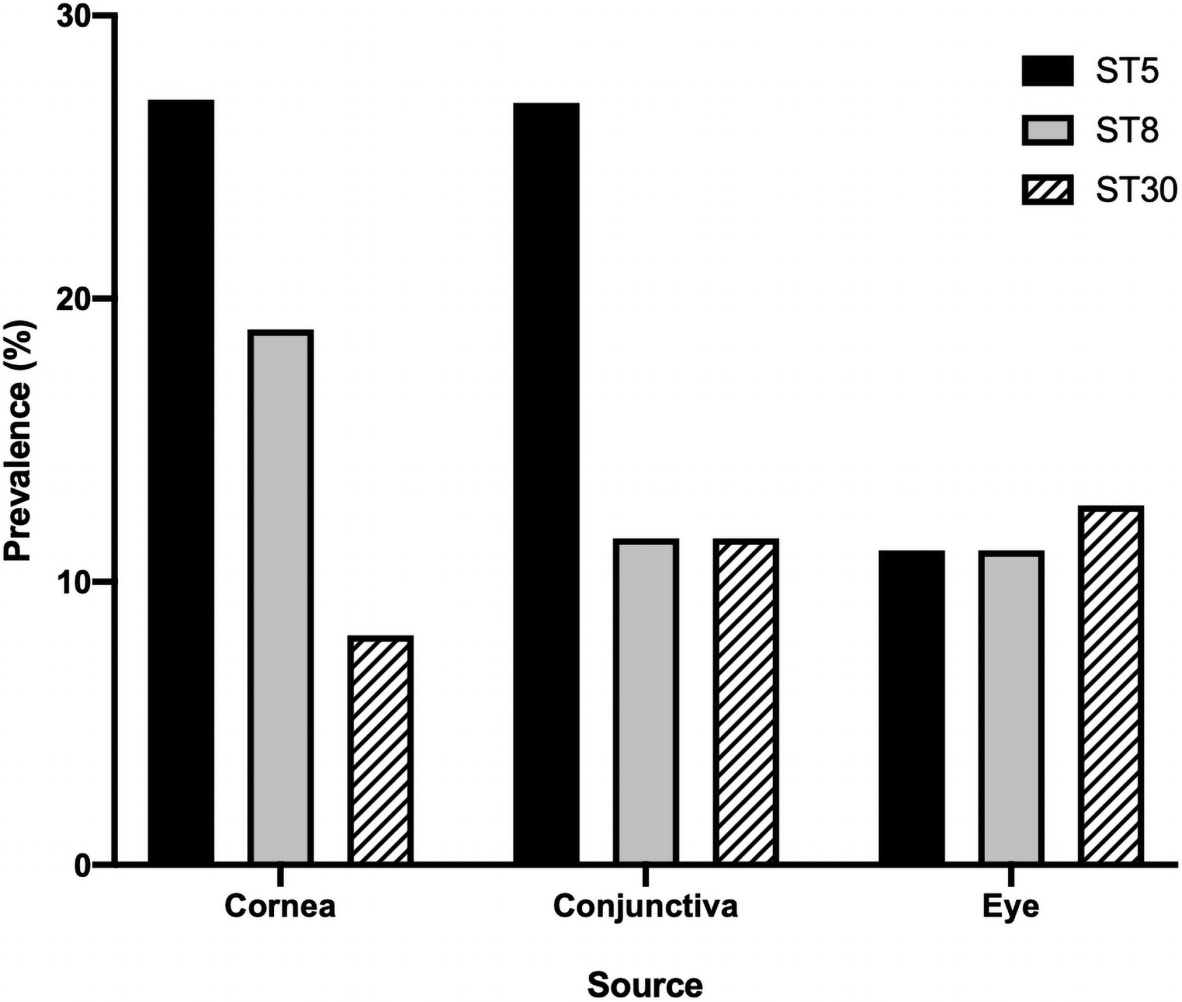
Prevalence of the three most abundant multilocus sequence types (MLST) among ocular *S*. *aureus* isolates based on specific anatomic source.

In order to establish whether specific *S*. *aureus* lineages are associated with ocular infections, all isolates underwent WGS using next-generation Illumina technology and processed through a custom analytical pipeline (S1 Fig). As a first step, the sequence type (ST) for each isolate was defined using multi-locus sequence typing (MLST). In this widely used classification system, the genomic sequences of seven housekeeping genes (*arcC*, *aroE*, *glpF*, *gmk*, *pta*, *tpi*, and *yqiL*) are used to determine genetic relatedness based on a well-defined reference database in order to generate a sequence type. Among the 163-member ocular strain set, there were 41 individual STs identified, with 3 isolates having no assigned ST (Fig 1). The most commonly represented STs were ST 5 (n = 34, 21%), ST 8 (n = 24, 15%), and ST 30 (n = 17, 11%) (Fig 1). Among *S*. *aureus* corneal isolates (n = 74) there were 23 individual STs, with ST5 (n = 20, 27%), ST8 (n = 14, 19%) and ST30 (n = 6, 8%) identified most commonly. ST5, ST8 and ST30 were also the most commonly identified STs among conjunctival isolates as well as those strains broadly categorized as from “eyes” (Fig 2).

#### Geography

**Fig 3:**
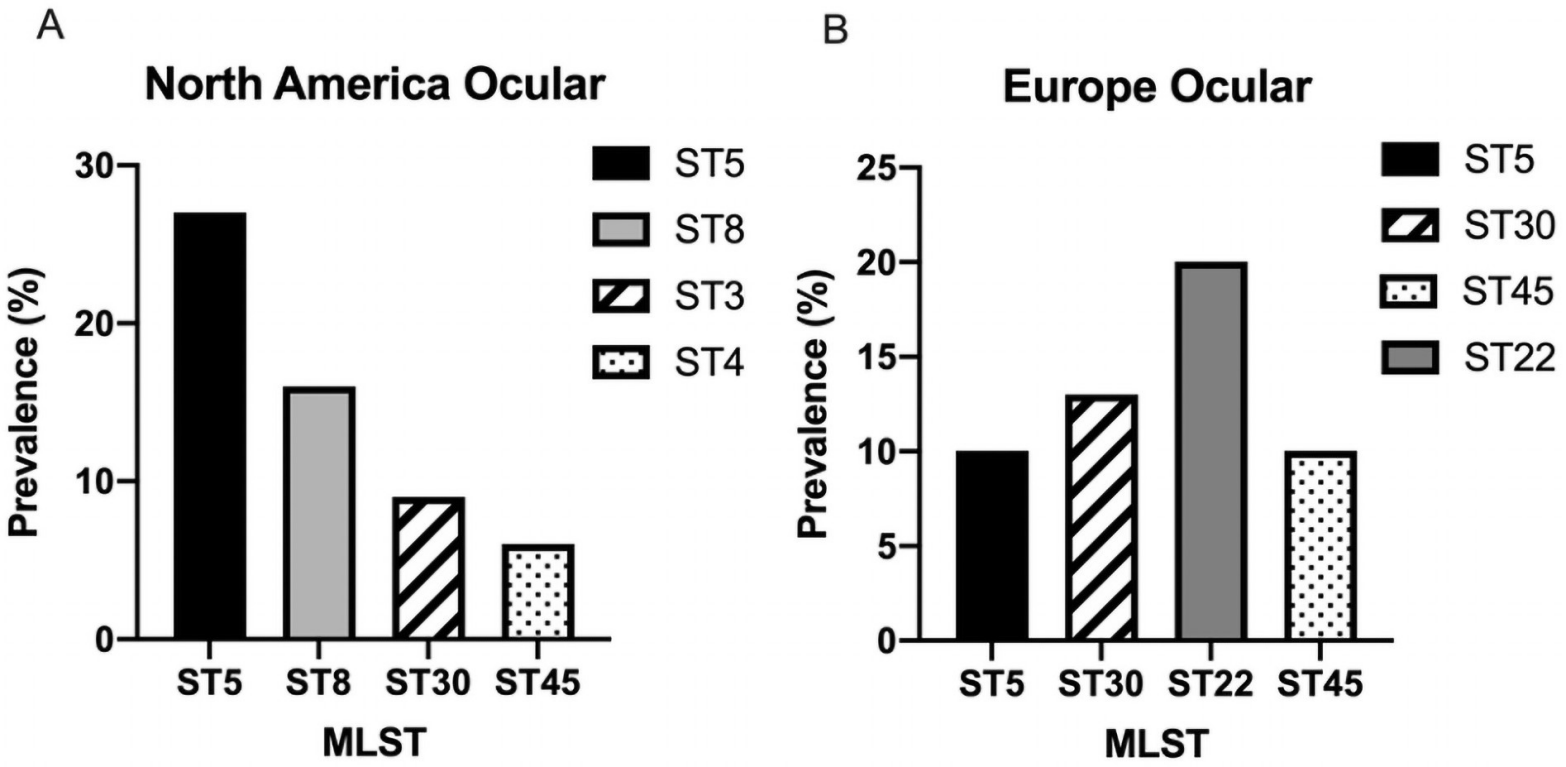
Prevalence of the most common multilocus sequence types (MLST) of ocular *S*. *aureus* isolates from North America (A) and Europe (B).

Among North American *S*. *aureus* ocular isolates (n = 110), 30 individual STs were identified, and reflecting the overall distribution of STs, the majority of isolates were categorized as either ST5 (n = 29, 27%), ST8 (n = 17, 16%), ST30 (n = 10, 9%) or ST45 (n = 6, 6%) (Fig 3A). Among the 30 ocular isolates from Europe, there were 16 individual STs with ST 22 (n = 6, 20%), ST30 (n = 4, 13%), ST5 (n = 3, 10%) and ST45 (n = 3, 10%) the most common (Fig 3B). There were far fewer ocular isolates collected from South America and the Middle East, but among the nine isolates from South America, ST8 (n = 4, 44%) was the most common out of 6 individual STs, while in the Middle East (n = 8), ST30 (n = 2, 25%) was the most prevalent out of 8 individual STs (S2 Fig).

#### Methicillin resistance

In the entire *S*. *aureus* ocular strain set, 50 isolates were previously classified as methicillin-resistant (MRSA) and 113 methicillin-sensitive (MSSA) based on minimum inhibitory concentration testing to oxacillin [47]. There was considerably more diversity of STs among MSSA strains with 37 individual strain types represented. However, ST30 (n = 15, 13%), ST5 (n = 14, 12%) and ST8 (n = 10, 9%) remained the most common STs among MSSA isolates. In contrast, among MRSA isolates there were only 13 individual STs, with 67% belonging to ST5 (n = 20, 39%) and ST 8 (n = 14, 27%). SCCmec typing which can further define MRSA lineages based on the genetic structure of the staphylococcal cassette chromosome *mec*, revealed 42% of ocular MRSA isolates to be SCC*mec* type II, 32% SCC*mec* type IVa, followed by IVc (12%), VIII (6%), I (4%) and IV (4%) (S1 Table).

#### Alternative strain typing methods

*agr typing*. Virulence factor expression in *S*. *aureus* is driven by a complex, intricate network of regulators that serve to coordinate environmental cues with a bacterial response. Among the well described global virulence factor regulators in *S*. *aureus*, the accessory gene regulator (*agr*) gene cluster encoding a peptide quorum sensing system, has known genetic variants circulating among *S*. *aureus* clinical isolates. Four distinct groups have been defined based on the production and response (inducing vs inhibition) to specific autoinducing peptides (AIPs). *agr* groupings are frequently correlated with MLST but there has also been evidence of individual *agr* groups associated with specific clinical lineages [48, 49]. Among the ocular strain set, 70 (43%) isolates were classified in *agr* group 1, 51 (31%) in group 2, 28 (17%) in group 3 and 14 (9%) in group 4 (S1 Table). There was a significant correlation between the MLST and *agr* type (p < 2.2e-16). For example, ST8 isolates were exclusively identified as *agr* type 1, all ST5 isolates were in *agr* type 2, and all ST30 isolates were found in *agr* type 3. As expected, *agr* typing did not significantly alter lineage classification compared to MLST among ocular isolates in this strain set.

*spa typing*. In addition to the MLST and *agr* typing classification systems, the genetic sequence of Staphylococcal Protein A (*spa*), an immunoglobulin-binding protein that acts to inhibit phagocytosis as well as promote host immune cell death [50], has also been widely used to type *S*. *aureus* clinical isolates [51, 52]. The *spa* gene contains three distinct regions, including a polymorphic X region that includes a 24 bp variable number tandem repeat (VNTR) region. Thus, a strain can be assigned a *spa* type based on the unique sequence and number of repeats within this polymorphic region. While it has been shown that *spa* typing often tracks with multi-locus sequence type, there can be meaningful differences that provide additional insight into the epidemiology of *S*. *aureu*s isolates. In the 163-member ocular *S*. *aureus* strain set, there 83 individual *spa* types identified with 11 strains having an unidentified type (S1 Table). The most commonly identified *spa* types included t002 (n = 20, 12%), t008 (n = 15, 9.2%) and t084 (n = 6, 3.7%). As expected, *spa* types tracked largely with MLST data. For example, among 34 ST5 isolates, while there were 10 individual *spa* types identified, 19 (56%) belonged to t002. Similarly, among 24 ST8 strains, there were 8 individual identified *spa* types with 2 additional unknown types, yet 12 (50%) were classified as *spa* type t008. Interestingly, there was more diversity among the 17 ST30 isolates with 12 individual *spa* types identified. t012 (n = 3, 18%) and t021 (n = 3, 18%) were the most commonly identified among this group. Overall, *spa* typing revealed greater diversity among ocular *S*. *aureus* strains compared to MLST or *agr* typing, demonstrating additional genetic differences between closely related strains.

### *S*. *aureus* virulence factors

Through multiple strain typing methods (MLST, *spa*, *agr*) we have identified several common circulating lineages associated with ocular infections. However, given the limited genetic context on which these typing methods rely, we sought to leverage the WGS data to explore additional genetic determinants to further characterize this ocular isolate set. *S*. *aureus* is known to encode a sizable and diverse arsenal of virulence factors that enable this organism to exploit a variety of environmental niches. In order to determine the association between common circulating lineages and the distribution of virulence factors, 235 individual virulence factors previously described to be associated with any type of *S*. *aureus* infection were compiled into a virulence factor database (S3 Table). This included 17 cytotoxin/hemolysins, 22 cellular adherence factors, 7 biofilm-associated factors, 11 proteases, 53 cell-wall associated proteins, 43 immune modulators/superantigens, 33 iron-scavenging and metabolism related factors, and 49 secreted toxins and associated secretory machinery. In order to include all strains in the analysis for the presence/absence of each factor, a loose criteria of 60% percent identity to the reference sequence was utilized.

**Fig 4:**
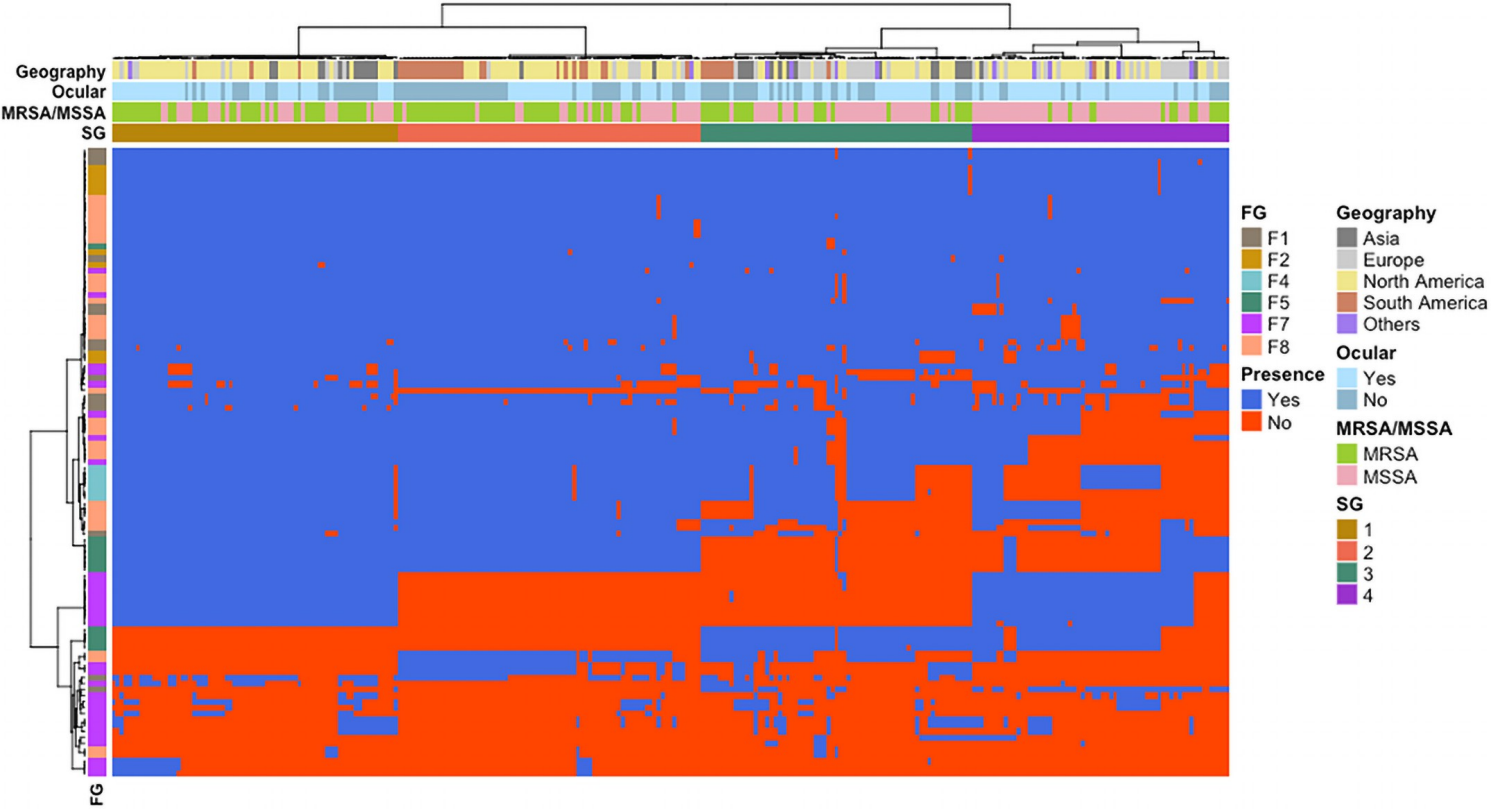
Heatmap plot demonstrating the pattern of virulence factor distribution across both ocular and non-ocular isolates. Using a Ward’s minimum variance method, the phylogenetic tree across the top was created based on the presence/absence of 235 known *S*. *aureus* virulence factors, while the tree on the left-hand side is a representation of virulence factor similarity. The geographical origin of the isolates, MRSA/MSSA status as well as ocular vs non-ocular designation is represented in rows. Conversely, columns represent individual virulence factors with functional groups (FG): F1—Adherence; F2—Cytotoxin/hemolysin; F4—Protease inhibitor; F5—Capsular polysaccharide; F7—Immune modulator; F8—Toxin.

Of the 235 virulence factors tested, 160 were found in over 95% of all ocular isolates tested indicating broad distribution of these genetic determinants. However, of the remaining 75 virulence factors, 49 were found in 50–94% of isolates and 26 were identified in less than 50% of strains. In order to further understand the distribution of these virulence factors across the ocular strain set, we sought to determine if there was an association between specific virulence factors and strain lineages (i.e., MLST). Due to the high overall number as well as the relative uneven distribution of sequence types found in our strain set (44 individual STs, 22 of which were only identified once), statistical analyses on MLST strain types were very limited. Thus, a clustering analysis was used to group similar STs together based on their overall virulence factor signature into strain groups (SGs) (Fig 4, S1 and S4 Tables). The statistically determined optimal number of groups was four, and this was supported by the fact that each of the major STs in this study (5, 8 and 30) clustered into separate SGs. For example, SG1 was comprised of 41 total isolates, including all ST5 strains (n = 32) as well as ST105 (n = 1), ST111 (n = 1), ST1181 (n = 1), ST25 (n = 1) ST72 (n = 3) and 2 strains without an assigned ST. As such, SGs allowed for statistical analyses of virulence factors and closely related groups of STs to further assess the distribution of virulence factors across the ocular strain set.

Overall, there were 75 virulence factors that had a significant association with one or more of the defined SGs (S4 Table). Among these were 11 bacterial adherence proteins, 10 cell-wall associated proteins, 2 cytotoxins, 27 immune modulators/superantigens, 6 proteases and 19 secreted toxins. Cell-wall associated capsular polysaccharide genes were found clustered together with *cap5H-K*, found in 100% of strains in SG1 and SG2 (groups that are primarily comprised of ST5 and 8, respectively), yet in only 15% of strains from SG3 (primarily comprised of ST239 and ST15) and 31% of strains in SG4 (primarily comprised of ST30 and ST45) (p = 3.28e-61). Conversely, *cap8H-K*, were found in 99% and 69% of SG3 and SG4 strains, respectively, and 0% of SG1 or SG2 strains (p = 3.28e-61). Other factors such as a set of enterotoxins typically associated with enterotoxin gene cluster 1 (*egc*) were found in 100% of strains in SG1 and 86% within SG4, with significantly fewer found among SG2 (1–3%) or SG3 (0%) isolates. Taken together, this data demonstrates that while the majority of known *S*. *aureus* virulence factors are found in all ocular isolates queried, there is a subset that are strongly associated with the previously identified common lineages (ie. ST5, 8, 30), suggesting that there may be specific virulence factors of particular importance in driving ocular infections.

### Ocular vs non-ocular isolates

In order to determine if those virulence factors with an association to a specific strain group are relevant to ocular infections or merely a reflection of general pathogenic *S*. *aureus* isolates, a comparative analysis of ocular vs non-ocular *S*. *aureus* clinical isolates was performed. As such, 116 non-ocular *S*. *aureus* isolates with fully sequenced and annotated, genomes were curated from NCBI to serve as a comparator strain set (Table 1, S2 Table). While two isolates dated back to 1884, the majority of isolates were collected from 1983–2015 (n = 110), with 95 of these isolated from 2000–2015. As with the ocular strain set, there was broad geographic representation among the non-ocular *S*. *aureus* strain set with 31 isolates (27%) from Asia, 30 (26%) from South America, 28 (24%) from Europe, 25 (22%) from North America, 1 (1%) from Africa, and 2 (2%) in which the continent of origin was unknown. The gender was only known for 19 isolates, and among these, 15 were from male patients, and 4 from female patients. Four isolates were collected from non-human sources. Age of the patient was only known for 33 isolates, but ranged from 0–87, with the majority from patients aged 60–79 (n = 10, 9%). Of the 112 human non-ocular *S*. *aureus* isolates, a source was recorded for 82 with the majority from wound/soft tissue infections (n = 31, 27%), bacteremia (n = 21, 19%) and nasal colonization (n = 14, 12%) (Table 1, S2 Table). 32 (28%) were classified as MSSA and 84 (72%) were classified as MRSA.

With respect to MLST, the non-ocular *S*. *aureus* strain set was comprised of 32 individual STs. Similar to the ocular isolates, ST 8 (n = 37, 32%) and ST 5 (n = 16, 14%) were among the most commonly identified strain type (S3 Fig). Among isolates derived from skin and soft tissue infections (n = 31), there were 16 individual STs with ST8 (n = 12, 39%), ST5 (n = 3, 10%) and ST239 (n = 3, 10%) the most commonly identified. Similarly, there were 9 individual STs among 22 isolates derived from blood (sepsis/bacteremia), with ST5 (n = 5, 23%), ST8 (n = 5, 23%) and ST239 (n = 5, 23%) most common, and 6 individual STs among 14 nasal isolates with ST 8 (n = 7, 50%), ST59 (n = 2, 14%) and ST239 (n = 2, 14%) the most common. ST8 and ST5 were found most commonly among both MRSA (ST8 38%, ST5 14%) and MSSA (ST8 25%, ST5 12.5%) non-ocular isolates. SCC*mec* typing of MRSA isolates revealed the majority of strains were classified as SCC*mec*IVa (26%), SCC*mec*II (11%) or SCC*mec*III (10%). Taken together, there were considerable similarities with respect to ST prevalence between the non-ocular and ocular strain sets demonstrating circulating ocular *S*. *aureus* isolates appear to be closely related to circulating non-ocular *S*. *aureus* strains, and establishing the non-ocular strain set as an appropriate comparator group for further analysis.

To establish whether particular virulence factors were enriched among ocular vs non-ocular isolates, the presence/absence of all 235 virulence factors were determined for the 116 non-ocular strains and the pattern of virulence factor distribution was compared to ocular isolates. Overall, a difference between ocular and non-ocular isolates was evident (p<1e-6) (Fig 4, S4 Fig), suggesting that there may be specific virulence factors associated with either ocular or non-ocular strains. On further analysis, while controlling for MRSA vs MSSA status as well as geographic origin in logistic regression models, there were 12 individual virulence factors that were significantly enriched among ocular isolates as compared to non-ocular strains (Table 2). These included10 secreted enterotoxin superantigens (SAgs) (*seu*, *selo*, *seln*, *selm*, *seg*, *selv*, *sei*, *sed*, *sej*, *ser*) and 2 enterotoxin pseudogenes (*Ψent1*, *Ψent2*). *Sed*, *sej* and *ser* were only found among ocular isolates, although these factors were present in approximately 13% of ocular isolates. The remaining virulence factors, *Ψent1*, *Ψent2*, *seu*, *selo*, *seln*, *selm*, *sei*, *seg*, *selv*, were found among ocular isolates at nearly 2-fold higher rates (55–57%) compared to non-ocular strains (30%) (p = .00184). Conversely, there were only four virulence factors that were identified as enriched among non-ocular isolates (Table 2), including 1 cellular adhesion gene (*fnbB*), 2 cell wall surface anchors (*sasG*, *sraP*) and 1 immune modulators/superantigens (*ssl8*). While *fnbB*, *sraP* and *ssl8* were found at rates less than 1.5-fold in non-ocular vs ocular isolates, *sasG* was found 4.3-fold higher (p = 2.02e-7) among non-ocular isolates.

**Table 2:** Virulence factors enriched among ocular and non-ocular strains, controlling for both MRSA/MSSA status and geography of strain origin.

*Gene*	*Function*	*Proportion Ocular*	*Proportion Non-ocular*	*Adj p-value (BH)*
*Ψ-ent2*	Enterotoxin	0.571	0.298	1.84E-02
*Ψ-ent1*	Enterotoxin	0.564	0.298	1.84E-02
*selo*	Enterotoxin	0.577	0.298	1.84E-02
*seln*	Enterotoxin	0.564	0.298	1.84E-02
*selm*	Enterotoxin	0.571	0.298	1.84E-02
*sei*	Enterotoxin	0.571	0.298	1.84E-02
*seg*	Enterotoxin	0.552	0.289	1.84E-02
*selv*	Enterotoxin	0.571	0.298	1.84E-02
*seu*	Enterotoxin	0.564	0.298	1.84E-02
*sed*	Enterotoxin	0.123	0	1.84E-02
*sej*	Enterotoxin	0.129	0	1.84E-02
*ser*	Enterotoxin	0.135	0	1.84E-02
*fnbB*	Cellular adhesion	0.779	0.930	1.84E-02
*sasG*	Cell wall surface anchor	0.166	0.711	2.02E-07
*sraP*	Cell wall surface anchor	0.791	0.947	2.70E-02
*ssl8*	Superantigen	0.761	0.956	4.49E-02

Among the SAgs identified as enriched in ocular isolates, 7 are known members of the enterotoxin gene cluster 1 (*egc1*), an operon first described to contain *selo*, *selm*, *sei*, *Ψent1*, *Ψent2*, *seln* and *seg* [37]. Several other *egc* variants have emerged as a result of genetic recombination and deletion events, including *egc2* which contains *seu* instead of the two pseudogenes [53]. Additionally, these enterotoxins are also often associated with mobile, *S*. *aureus* pathogenicity islands (SaPIs). As demonstrated in Table 3, the enterotoxin gene cluster containing *selo*, *selm*, *sei*, *seln* and *seg* were found with nearly equal distribution between *egc1* and *egc2* and both of these gene clusters were found to be primarily associated with either SapI2 (37%) or SapI3 (50%). Further analysis demonstrated that both *egcs* were highly conserved among these strains (data not shown), and of the 40 strains containing *egc1*, 82% of these were associated with SapI3, and conversely, of the 53 strains containing *egc2*, 53% were associated with SaPI2. Other enterotoxins (*ser*, *selv*, *sed*, *sej*) were not found associated with an enterotoxin gene cluster, yet 100% of these genes were still found among SaPIs, with the majority associated with SapI3. Thus, this data demonstrates the clear association between this set of enriched virulence with mobile genetic elements, suggesting a mechanism for horizontal gene transfer and dissemination of genes that may portend a competitive advantage in ocular infections.

**Table 3:** Genetic location of enterotoxins found to be significantly enriched among 163 ocular *S*. *aureus* isolates.

Virulence factor	# of isolates	% SaPIn1	% SaPI2	% SaPI3	% *egc1*	% *egc2*
*ψent1*, *ψent2*	40	2	15	83	100	-^a^
*selo*	94^b^	13	37	50	44	56
*seln*	93	13	37	50	44	56
*selm*	93	13	37	50	44	56
*sei*	93	13	37	50	44	56
*seg*	93	13	37	50	44	56
*seu*	52	22	54	25	-^c^	100
*ser*	19	5	25	70	-^d^	-^d^
*selv*	58	0	36	64	-^d^	-^d^
*sed*	19	5	20	75	-^d^	-^d^
*sej*	19	5	26	68	-^d^	-^d^

^a^Pseudogenes ψent1 and ψent2 occur on egc1 only.

^b^One isolate carried *selO* only.

^c^s*eu* is present on egc2 only.

^d^These enterotoxins are not associated with any described egcs.

## Discussion

In this study, the analysis of whole genome sequencing data derived from a diverse set of contemporary ocular *S*. *aureus* isolates has simultaneously defined common circulating ocular strain lineages as well as provided an unbiased approach to identify virulence factors enriched among ocular isolates. With respect to MLST typing, we have shown that circulating ocular *S*. *aureus* clinical isolates are predominantly ST 5 (n = 34, 21%), ST 8 (n = 24, 15%), and ST 30 (n = 17, 11%). This aligns nearly identically with the recent report of 56 ocular isolates collected from Massachusetts Eye and Ear Infirmary that demonstrated 23.2% ST5, 16% ST8 and 11% ST30 [13]. Similarly, a set of 75 *S*. *aureus* keratitis isolates collected from the University of Miami, Bascom Palmer Eye Institute, included 40% isolates of clonal complex (CC) 5 (ST5 is classified within CC5), and 37.3% of CC8 (ST8 is classified within CC8) [16]. Interestingly, only one isolate from this study set was identified in clonal complex 30. In contrast, among 90 MRSA ocular isolates from a tertiary care hospital in South India, ST772 and ST22 were found most commonly, with only one isolate identified as ST8, 4, or ST30, and none as ST5 [14], reflecting potentially important regional differences in circulating ocular *S*. *aureus* isolates.

Overall, while it is not known what percent of the ocular strains are community-acquired vs hospital-acquired, the predominance of ST5 and ST8 as well as *spa* types t002 and t008 strains among ocular isolates found in this study mirrors the predominant circulating non-ocular *S aureus* isolates in the United States and Europe [54]. ST8 MRSA strains from North America, also classified as USA300 (a designation based on pulsed-field gel electrophoresis typing), have emerged over the past 20 years as the most common cause of community acquired skin and soft tissue MRSA infections in the United States [55, 56]. Similarly, ST5 MRSA isolates which include the USA800 and USA100 clones have been identified as most prevalent cause of hospital-acquired infections in North and South America [57]. Studies focused on the epidemiology of *spa* types among circulating *S*. *aureus* isolates have also identified similar trends with t002 (ST5) and t008 (ST8) as consistently among the most common circulating *spa* types in North America as well as Europe [58–61].

Taken together, from a traditional lineage classification standpoint, the *S*. *aureus* strains isolated from ocular infections appear to align well with the major circulating pathogenic *S*. *aureus* strains capable of causing disease in other organ systems. However, given the inherent genetic diversity of *S*. *aureus* and its success in a wide diversity of environmental niches, it seemed plausible that there would be key differences with respect to the acquisition of virulence factors between ocular and non-ocular isolates. In order to explore this concept further, our 163 ocular isolate collection was directly compared to a 113 non-ocular strain set for the presence/absence of 235 known virulence factors. The vast majority of virulence factors were distributed equally among ocular and non-ocular isolates, including previously studied *pvl* and *lukE* (both found in 100% of ocular and non-ocular strains using the criteria of at least 60% identity to a reference sequence) and *sea* (found in 23% of ocular and 14% of non-ocular strains). However, 13 were found to be significantly enriched among ocular isolates. Of these 13, 12 are considered enterotoxin superantigens (SAgs) (*ser*, *seJ*, *sed*, *seu*, *selv*, *seg*, *seI*, *selM*, *selN*, *selO*, *Ψent1*, *Ψent2*,) and one (*set26*) was a secreted exotoxin with homology to enterotoxins.

Enterotoxins are considered superantigens (SAgs) due to their ability to bind major histocompatibility complex class II (MHCII) molecules leading to widespread, non-specific T-cell activation and subsequent massive cytokine release [62]. SAgs are the key drivers of toxic shock syndrome, eliciting fever, hypotension and eventually end-organ failure [63] as well as promoting *S*. *aureus* food poisoning through potent emetic activity [64]. The role of enterotoxins in ocular infections remains to be fully defined, but they may play a role in immune modulation as well as corneal ulceration. For example, among *S*. *aureus* isolates collected from patients with various forms of allergic conjunctivitis, strains carrying enterotoxins were identified more frequently from patients with concurrent corneal ulceration compared to patients with no ulceration [65]. Additionally, Enterotoxin B (*seb*) has been shown to be toxic to corneal epithelial cells as well as induce changes in cytokine expression in an *in vitro* cell culture model [66]. Furthermore, treatment with *Staphylococcal* Enterotoxin B has been used to suppress immune rejection during murine corneal transplantation potentially due to its effects on T-cell depletion and host non-specific tolerance [67].

There is a growing number of described enterotoxins and enterotoxin-like genes identified in *S*. *aureus*, found both in the *S*. *aureus* chromosome as well as embedded in mobile genetic elements (MGEs) such as pathogenicity islands and enterotoxin gene clusters (*egcs*), and it is thought that upwards of 80% of all *S*. *aureus* isolates including both pathogenic and non-pathogenic isolates carry an average of 5–6 enterotoxin genes [37, 64, 68]. The canonical enterotoxin gene cluster (*egc1*) is comprised of 5 enterotoxin genes, *selo*, *selm*, *sei*, *seln*, *seg*, and two pseudogenes, *Ψent1 and Ψent2* [37]. While variants of *egc* have emerged as a result of recombination, addition and deletion events, several studies have examined the prevalence of *egc* among subsets of clinical isolates. For example, the *egc* cluster has been found in high percentage among cystic fibrosis lung isolates [69], *Staphylococcal* scarlet fever isolates, and furunculosis isolates [37], supporting a potential role for enterotoxins in these disease settings. Conversely, a lower prevalence of *egc* has been identified in nasal carriage isolates, endocarditis and septic shock [37, 70, 71]. Taken together, the role of enterotoxins in promoting *S*. *aureus* infections may be variable, depending on the specific disease setting. In the current study, either *egc1* or *egc2* was identified in nearly 60% of ocular isolates compared to 30% of non-ocular isolates. Additional enterotoxins, *selv*, *sed*, *sej*, and *ser* were also enriched among ocular isolates in this strain set and all enterotoxins identified were associated with mobile pathogenicity islands. While enterotoxins are clearly not essential for ocular tissue infections, given their disproportionate prevalence among ocular isolates, they may provide a competitive advantage. Given the location of these genes on mobile genetic elements, horizontal gene transfer could facilitate the acquisition of enterotoxins.

## Conclusion

Whole genome sequencing can provide a powerful tool to explore contemporary bacterial population structures with respect to circulating lineages as well as understanding genetic determinants associated with human disease. Here, we demonstrate that while ocular isolates appear to reflect commonly pathogenic *S*. *aureus* non-ocular isolates with respect to standard strain typing methods, on further analysis, there were important differences with respect to virulence factor distribution. Leveraging WGS data, it is possible to prospectively define potential *S*. *aureus* virulence factors or other genetic determinants that may promote infection in specific disease settings, thereby deepening our understanding of this important human pathogen.

## Supporting information

S1 Fig (TIFF)

S2 Fig Most common multilocus sequence type (MLST) among ocular isolates from South America and the Middle East. (TIFF)

S3 Fig goeBURST full minimum spanning tree of 116 *Staphylococcus aureus* non-ocular isolate multilocus sequence type (MLST) profiles. The three most abundant MLSTs in each group are shown in black, while all others are shown in grey. Nodes are scaled according to the abundance of each MLST, while connecting links are show in greyscale to represent the number of MLST allelic differences (range from 1 to 7) with darker links representing fewer differences.
(TIFF)

S4 Fig Principal coordinate Analysis (PCoA) with the Jaccard distance. Symbols indicate ocular vs non-ocular isolates. Colors represent individual strain groups. Ellipses represent 95% confidence ellipsoids for ocular and non-ocular samples.
(TIFF)

S1 Table Date of isolation, anatomic source, geographic region, *agr* type, multilocus sequence type (MLST), *spa* type, and methicillin resistance status, SCC*mec* type, and strain group of the 163 *Staphylococcus aureus* ocular isolates used in this study. (DOCX)

S2 Table Source, date of isolation, geographic region, multilocus sequence type (MLST), methicillin resistance status, and SCC*mec* type of the 116 *Staphylococcus aureus* non-ocular isolates used in this study. References are given for each isolate when available, with a dash indicating that no publicly available citation for the genome of an isolate could be identified.
(DOCX)

S3 Table Class, gene identity, function, and reference genome of the 235 *Staphylococcus aureus* virulence factor database. (DOCX)

S4 Table Virulence factors associated with one or more strain groups (SGs). (DOCX)
