# Supplementary figures and images for: Genomics of Staphylococcus aureus ocular isolates
Source: PLoS One. 2021 May 3;16(5):e0250975. doi: 10.1371/journal.pone.0250975 (PMC8092774; doi:10.1371/journal.pone.0250975)

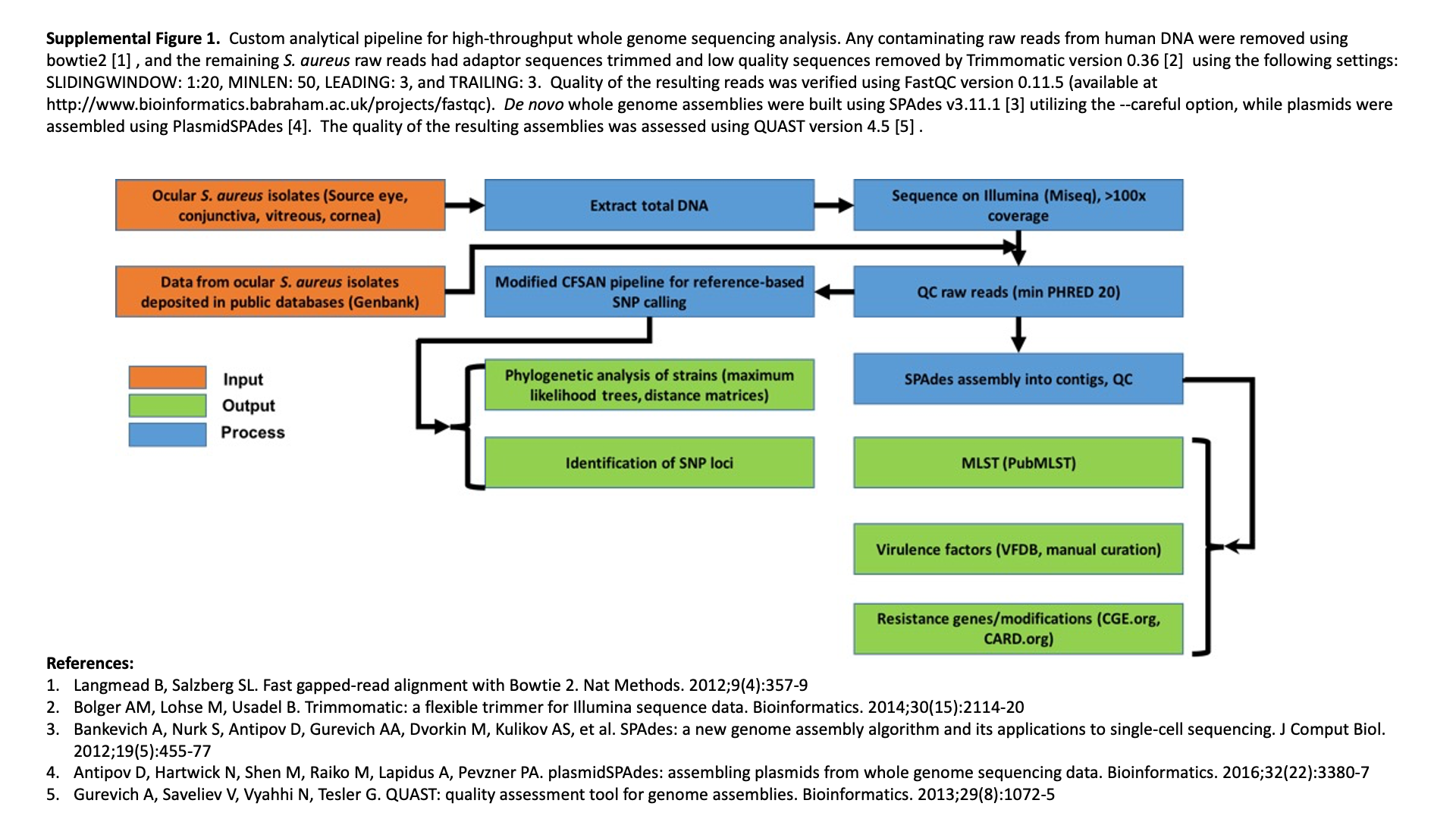

Supplement: S1 Fig — (TIFF) [file pone.0250975.s001.tiff]

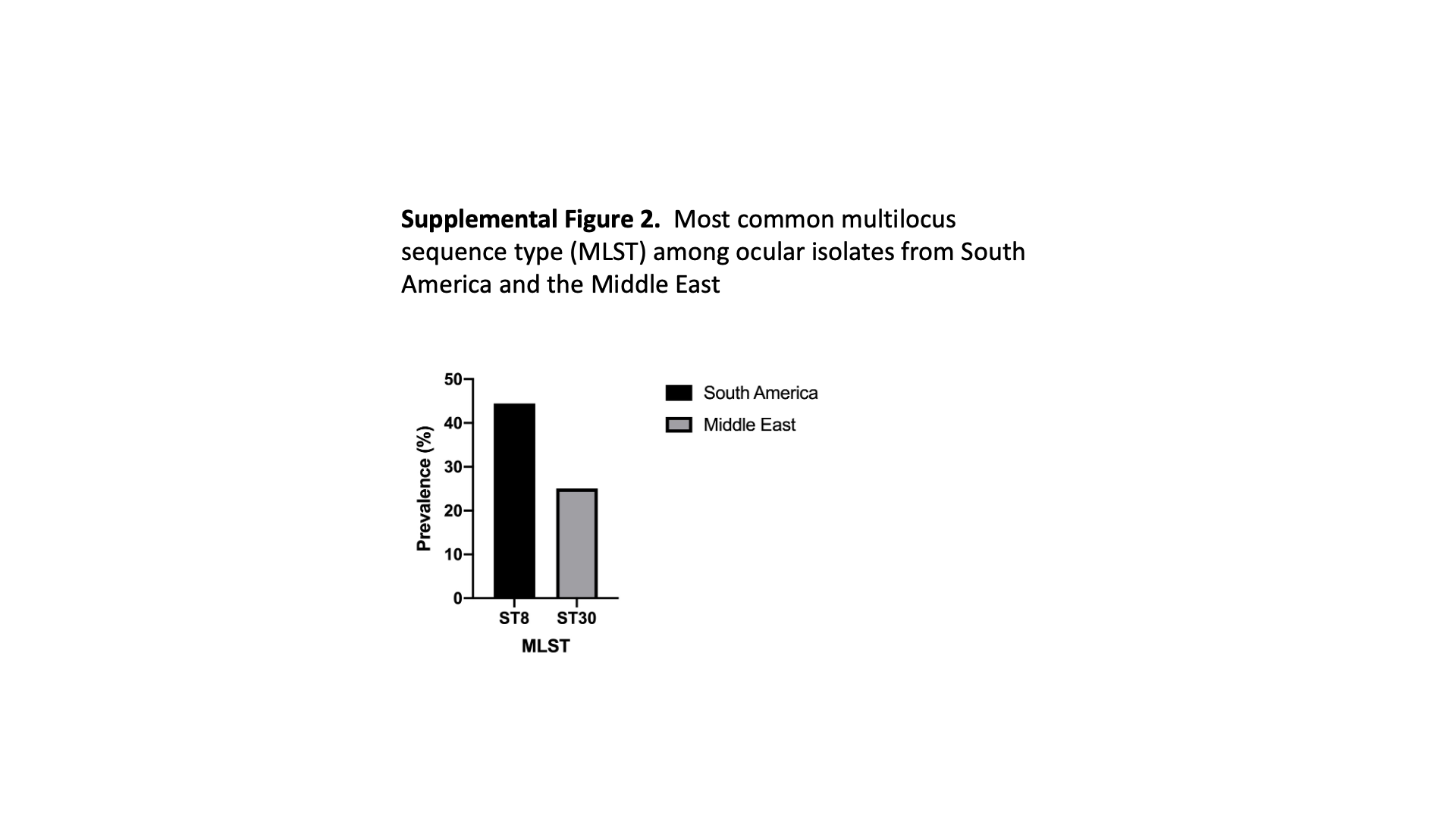

Supplement: S2 Fig — (TIFF) [file pone.0250975.s002.tiff]

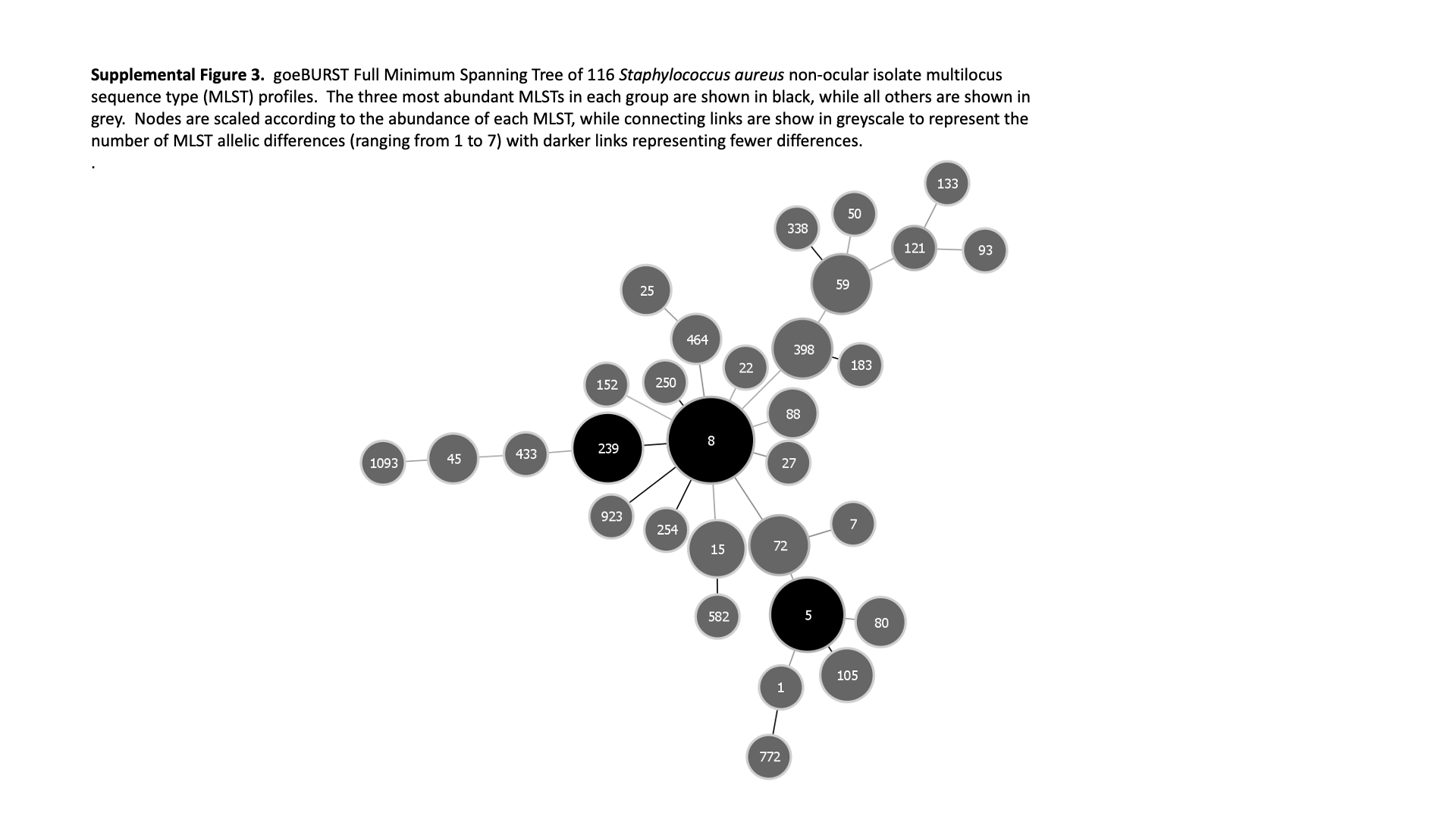

Supplement: S3 Fig — The three most abundant MLSTs in each group are shown in black, while all others are shown in grey. Nodes are scaled according to the abundance of each MLST, while connecting links are show in greyscale to represent the number of MLST allelic differences (range from 1 to 7) with darker links representing fewer differences. (TIFF) [file pone.0250975.s003.tiff]

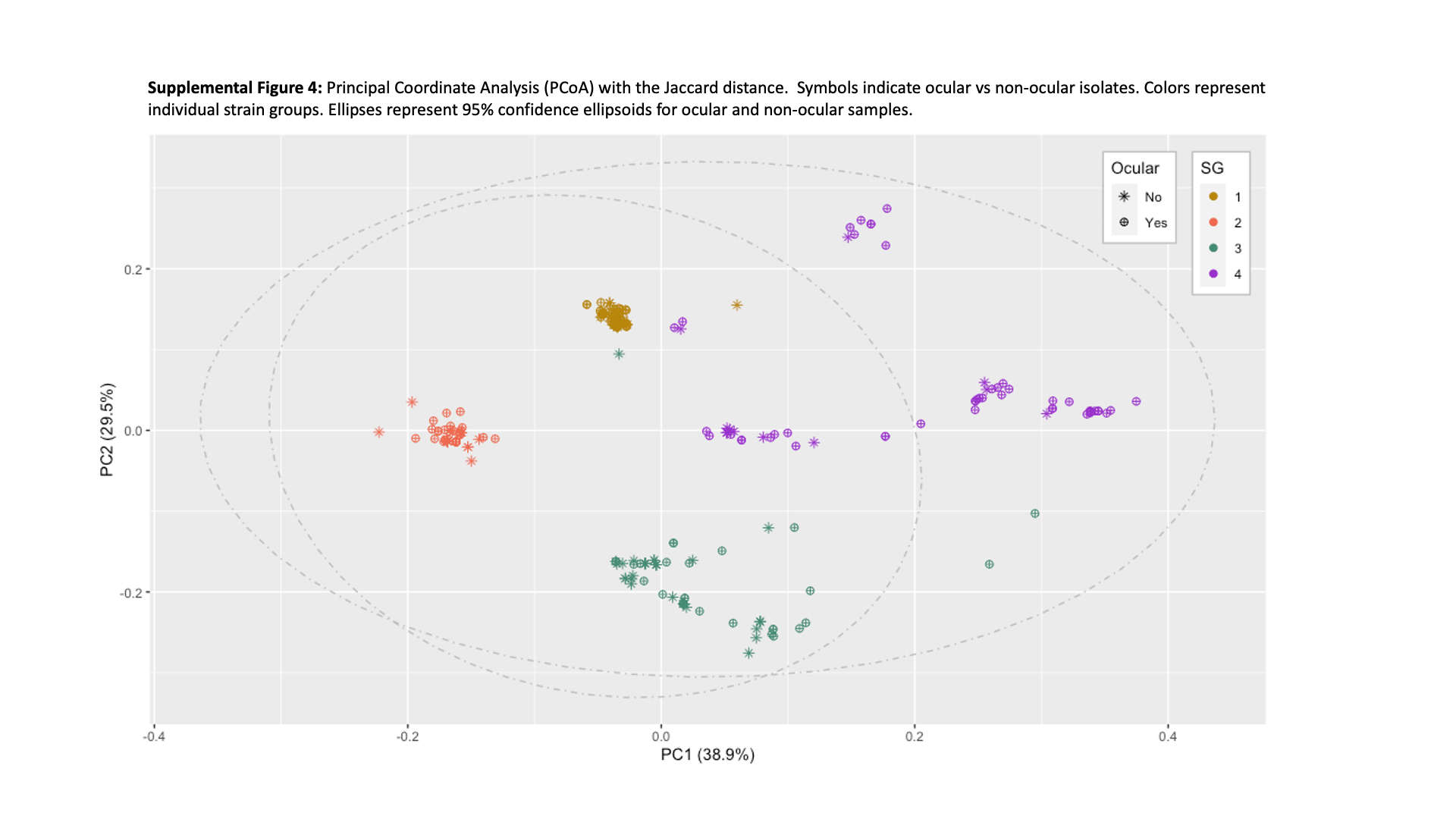

Supplement: S4 Fig — Symbols indicate ocular vs non-ocular isolates. Colors represent individual strain groups. Ellipses represent 95% confidence ellipsoids for ocular and non-ocular samples. (TIFF) [file pone.0250975.s004.tiff]
