# Supplementary material for: Genomics of Staphylococcus aureus ocular isolates
Source: PLoS One. 2021 May 3;16(5):e0250975. doi: 10.1371/journal.pone.0250975 (PMC8092774; doi:10.1371/journal.pone.0250975)
Supplement: S1 Table — (DOCX) [file pone.0250975.s005.docx]

| Isolate  **S1 Table**. Date of isolation, anatomic source, geographic region, *agr* type, multilocus sequence type (MLST), *spa* type, and methicillin resistance status, SCC*mec* type, and strain group of the 163 *Staphylococcus aureus* ocular isolates used in this study. |  | Date |  | Source |  | Region |  | *agr* type |  | MLST |  | *spa* type |  | MSSA/MRSA |  | SCC*mec* Type |  | Strain Group |  | Accession |
| --- | --- | --- | --- | --- | --- | --- | --- | --- | --- | --- | --- | --- | --- | --- | --- | --- | --- | --- | --- | --- |
| IHMA1 |  | 2008 |  | Eye |  | Asia |  | I |  | ST97 |  | t359 |  | MSSA |  | - |  | 2 |  | JADGYH000000000 |
| IHMA2 |  | 2008 |  | Eye |  | Asia |  | II |  | ST5 |  | t002 |  | MRSA |  | II |  | 1 |  | JADGVY000000000 |
| IHMA3 |  | 2008 |  | Eye |  | Asia |  | II |  | ST15 |  | t084 |  | MSSA |  | - |  | 3 |  | JADGVO000000000 |
| IHMA4 |  | 2009 |  | Eye |  | Asia |  | I |  | ST45 |  | t630 |  | MRSA |  | II |  | 4 |  | JADGVD000000000 |
| IHMA5 |  | 2009 |  | Eye |  | Asia |  | I |  | ST340 |  | t008 |  | MSSA |  | - |  | 2 |  | JADGUS000000000 |
| IHMA6 |  | 2009 |  | Eye |  | North America |  | I |  | ST8 |  | t008 |  | MSSA |  | - |  | 2 |  | JADGUH000000000 |
| IHMA7 |  | 2009 |  | Eye |  | North America |  | II |  | ST5 |  | t002 |  | MRSA |  | II |  | 1 |  | JADGTX000000000 |
| IHMA8 |  | 2009 |  | Eye |  | Asia |  | IV |  | ST121 |  | t3454 |  | MSSA |  | - |  | 4 |  | JADGTN000000000 |
| IHMA9 |  | 2010 |  | Eye |  | Asia |  | II |  | ST2126 |  | t084 |  | MSSA |  | - |  | 3 |  | JADGTD000000000 |
| IHMA10 |  | 2010 |  | Eye |  | South America |  | III |  | ST30 |  | t046 |  | MRSA |  | IVa |  | 4 |  | JADGYG000000000 |
| IHMA11 |  | 2012 |  | Eye |  | Asia |  | III |  | ST30 |  | t11029 |  | MSSA |  | - |  | 4 |  | JADGXV000000000 |
| IHMA12 |  | 2010 |  | Eye |  | Asia |  | III |  | ST88 |  | t5562 |  | MSSA |  | - |  | 3 |  | JADGXK000000000 |
| IHMA13 |  | 2012 |  | Eye |  | North America |  | I |  | ST8 |  | t008 |  | MRSA |  | IVa |  | 2 |  | JADGWZ000000000 |
| IHMA14 |  | 2012 |  | Eye |  | Asia |  | III |  | ST30 |  | t019 |  | MRSA |  | IVc |  | 4 |  | JADGWO000000000 |
| IHMA15 |  | 2012 |  | Eye |  | South America |  | I |  | ST395 |  | t7827 |  | MSSA |  | - |  | 3 |  | JADGWD000000000 |
| IHMA16 |  | 2014 |  | Eye |  | Europe |  | II |  | ST582 |  | t084 |  | MSSA |  | - |  | 3 |  | JADGWC000000000 |
| IHMA17 |  | 2014 |  | Eye |  | South America |  | I |  | ST8 |  | t846 |  | MRSA |  | IVc |  | 2 |  | JADGWB000000000 |
| IHMA18 |  | 2014 |  | Eye |  | South America |  | I |  | ST8 |  | t024 |  | MSSA |  | - |  | 2 |  | JADGWA000000000 |
| IHMA19 |  | 2014 |  | Eye |  | North America |  | I |  | ST8 |  | t008 |  | MRSA |  | IVa |  | 2 |  | JADGVZ000000000 |
| IHMA20 |  | 2014 |  | Conjunctiva |  | North America |  | II |  | ST5 |  | t893 |  | MSSA |  | - |  | 1 |  | JADGVX000000000 |
| IHMA21 |  | 2014 |  | Conjunctiva |  | North America |  | II |  | ST5 |  | t002 |  | MRSA |  | II |  | 1 |  | JADGVW000000000 |
| IHMA22 |  | 2014 |  | Conjunctiva |  | North America |  | II |  | ST5 |  | t688 |  | MSSA |  | - |  | 1 |  | JADGVV000000000 |
| IHMA23 |  | 2014 |  | Cornea |  | North America |  | II |  | ST15 |  | t084 |  | MSSA |  | - |  | 3 |  | JADGVU000000000 |
| IHMA24 |  | 2014 |  | Conjunctiva |  | North America |  | II |  | ST5 |  | t002 |  | MRSA |  | II |  | 1 |  | JADGVT000000000 |
| IHMA25 |  | 2014 |  | Eye |  | Europe |  | IV |  | ST50 |  | t246 |  | MSSA |  | - |  | 4 |  | JADGVS000000000 |
| IHMA26 |  | 2014 |  | Conjunctiva |  | North America |  | II |  | ST15 |  | t364 |  | MSSA |  | - |  | 3 |  | JADGVR000000000 |
| IHMA27 |  | 2014 |  | Cornea |  | North America |  | I |  | ST8 |  | t008 |  | MRSA |  | II |  | 2 |  | JADGVQ000000000 |
| IHMA28 |  | 2014 |  | Eye |  | Europe |  | II |  | ST5 |  | t777 |  | MRSA |  | VIII |  | 1 |  | JADGVQ000000000 |
| IHMA29 |  | 2014 |  | Cornea |  | North America |  | II |  | ST15 |  | t2556 |  | MSSA |  | - |  | 3 |  | JADGVP000000000 |
| IHMA30 |  | 2014 |  | Eye |  | North America |  | III |  | ST30 |  | t7110 |  | MSSA |  | - |  | 4 |  | JADGVN000000000 |
| IHMA31 |  | 2014 |  | Cornea |  | North America |  | II |  | ST5 |  | t002 |  | MRSA |  | II |  | 1 |  | JADGVM000000000 |
| IHMA32 |  | 2014 |  | Eye |  | Europe |  | II |  | ST5 |  | t045 |  | MRSA |  | I |  | 1 |  | JADGVL000000000 |
| IHMA33 |  | 2014 |  | Eye |  | Europe |  | I |  | ST8 |  | t024 |  | MRSA |  | IVc |  | 2 |  | JADGVK000000000 |
| IHMA34 |  | 2014 |  | Conjunctiva |  | North America |  | IV |  | ST59 |  | t216 |  | MSSA |  | - |  | 3 |  | JADGVJ000000000 |
| IHMA35 |  | 2014 |  | Cornea |  | North America |  | IV |  | ST87 |  | t316 |  | MSSA |  | - |  | 3 |  | JADGVI000000000 |
| IHMA36 |  | 2014 |  | Cornea |  | North America |  | I |  | ST45 |  | t330 |  | MSSA |  | - |  | 4 |  | JADGVH000000000 |
| IHMA37 |  | 2014 |  | Cornea |  | North America |  | I |  | ST8 |  | t008 |  | MSSA |  | - |  | 2 |  | JADGVG000000000 |
| IHMA38 |  | 2014 |  | Eye |  | North America |  | I |  | ST72 |  | t148 |  | MSSA |  | - |  | 1 |  | JADGVF000000000 |
| IHMA39 |  | 2014 |  | Conjunctiva |  | North America |  | III |  | ST34 |  | t166 |  | MSSA |  | - |  | 4 |  | JADGVE000000000 |
| IHMA40 |  | 2014 |  | Conjunctiva |  | North America |  | III |  | ST30 |  | t021 |  | MSSA |  | - |  | 4 |  | JADGVC000000000 |
| IHMA41 |  | 2014 |  | Cornea |  | North America |  | I |  | ST6 |  | t1476 |  | MSSA |  | - |  | 3 |  | JADGVB000000000 |
| IHMA42 |  | 2014 |  | Cornea |  | North America |  | II |  | ST5 |  | t002 |  | MSSA |  | - |  | 1 |  | JADGVA000000000 |
| IHMA43 |  | 2014 |  | Cornea |  | North America |  | III |  | ST30 |  | t021 |  | MSSA |  | - |  | 4 |  | JADGUZ000000000 |
| IHMA44 |  | 2014 |  | Cornea |  | North America |  | I |  | ST1181 |  | t334 |  | MSSA |  | - |  | 2 |  | JADGUY000000000 |
| IHMA45 |  | 2014 |  | Eye |  | Europe |  | III |  | ST30 |  | t046 |  | MSSA |  | - |  | 4 |  | JADGUX000000000 |
| IHMA46 |  | 2014 |  | Eye |  | North America |  | I |  | ST45 |  | t3776 |  | MSSA |  | - |  | 4 |  | JADGUW000000000 |
| IHMA47 |  | 2015 |  | Eye |  | North America |  | IV |  | ST87 |  | t471 |  | MSSA |  | - |  | 3 |  | JADGUV000000000 |
| IHMA48 |  | 2014 |  | Eye |  | North America |  | II |  | ST5 |  | t688 |  | MSSA |  | - |  | 1 |  | JADGUU000000000 |
| IHMA49 |  | 2014 |  | Eye |  | North America |  | II |  | ST5 |  | t688 |  | MSSA |  | - |  | 1 |  | JADGUT000000000 |
| IHMA50 |  | 2015 |  | Eye |  | Europe |  | II |  | ST5 |  | t002 |  | MRSA |  | IVc |  | 1 |  | JADGUR000000000 |
| IHMA51 |  | 2015 |  | Eye |  | Europe |  | I |  | ST22 |  | Unknown |  | MRSA |  | IV |  | 4 |  | JADGUQ000000000 |
| IHMA52 |  | 2015 |  | Cornea |  | North America |  | II |  | ST5 |  | t088 |  | MRSA |  | IVc |  | 1 |  | JADGUP000000000 |
| IHMA53 |  | 2015 |  | Eye |  | North America |  | I |  | ST672 |  | t3841 |  | MSSA |  | - |  | 4 |  | JADGUO000000000 |
| IHMA54 |  | 2015 |  | Eye |  | North America |  | II |  | ST105 |  | t002 |  | MRSA |  | II |  | 1 |  | JADGUN000000000 |
| IHMA55 |  | 2015 |  | Cornea |  | North America |  | II |  | ST5 |  | t105 |  | MSSA |  | - |  | 1 |  | JADGUM000000000 |
| IHMA56 |  | 2015 |  | Cornea |  | North America |  | II |  | ST5 |  | t002 |  | MRSA |  | II |  | 1 |  | JADGUL000000000 |
| IHMA57 |  | 2015 |  | Cornea |  | North America |  | I |  | ST8 |  | t008 |  | MRSA |  | IVa |  | 2 |  | JADGUK000000000 |
| IHMA58 |  | 2015 |  | Cornea |  | North America |  | I |  | ST8 |  | t121 |  | MRSA |  | IVa |  | 2 |  | JADGUJ000000000 |
| IHMA59 |  | 2015 |  | Cornea |  | North America |  | III |  | ST30 |  | t1487 |  | MSSA |  | - |  | 4 |  | JADGUI000000000 |
| IHMA60 |  | 2015 |  | Cornea |  | North America |  | II |  | ST5 |  | t002 |  | MSSA |  | - |  | 1 |  | JADGUG000000000 |
| IHMA61 |  | 2015 |  | Cornea |  | North America |  | II |  | ST5 |  | t045 |  | MRSA |  | II |  | 1 |  | JADGUF000000000 |
| IHMA62 |  | 2015 |  | Cornea |  | North America |  | I |  | ST45 |  | t362 |  | MRSA |  | IVa |  | 4 |  | JADGUE000000000 |
| IHMA63 |  | 2015 |  | Conjunctiva |  | North America |  | III |  | ST30 |  | t5074 |  | MSSA |  | - |  | 4 |  | JADGUD000000000 |
| IHMA64 |  | 2015 |  | Cornea |  | North America |  | II |  | ST333 |  | t279 |  | MSSA |  | - |  | 3 |  | JADGUC000000000 |
| IHMA65 |  | 2015 |  | Cornea |  | North America |  | II |  | ST5 |  | t002 |  | MSSA |  | - |  | 1 |  | JADGUB000000000 |
| IHMA66 |  | 2015 |  | Cornea |  | North America |  | II |  | ST5 |  | t688 |  | MRSA |  | II |  | 1 |  | JADGUA000000000 |
| IHMA67 |  | 2015 |  | Cornea |  | North America |  | I |  | ST8 |  | Unknown |  | MRSA |  | IVa |  | 2 |  | JADGTZ000000000 |
| IHMA68 |  | 2015 |  | Cornea |  | North America |  | I |  | ST398 |  | t1451 |  | MSSA |  | - |  | 4 |  | JADPXJ000000000 |
| IHMA69 |  | 2015 |  | Eye |  | Europe |  | I |  | ST22 |  | t1275 |  | MSSA |  | - |  | 4 |  | JADGTY000000000 |
| IHMA70 |  | 2015 |  | Cornea |  | North America |  | I |  | ST8 |  | t068 |  | MSSA |  | - |  | 2 |  | JADGTW000000000 |
| IHMA71 |  | 2015 |  | Cornea |  | North America |  | II |  | ST5 |  | t067 |  | MSSA |  | - |  | 1 |  | JADGTV000000000 |
| IHMA72 |  | 2015 |  | Conjunctiva |  | North America |  | III |  | ST30 |  | t363 |  | MSSA |  | - |  | 4 |  | JADGTU000000000 |
| IHMA73 |  | 2015 |  | Conjunctiva |  | North America |  | I |  | ST25 |  | t9374 |  | MSSA |  | - |  | 1 |  | JADGTT000000000 |
| IHMA74 |  | 2015 |  | Cornea |  | North America |  | I |  | ST8 |  | t008 |  | MSSA |  | - |  | 2 |  | JADPXH000000000 |
| IHMA75 |  | 2015 |  | Cornea |  | North America |  | II |  | ST5 |  | t002 |  | MSSA |  | - |  | 1 |  | JADGTS000000000 |
| IHMA76 |  | 2015 |  | Eye |  | Europe |  | III |  | ST4618 |  | t122 |  | MSSA |  | - |  | 4 |  | JADGTR000000000 |
| IHMA77 |  | 2015 |  | Cornea |  | North America |  | II |  | ST5 |  | t002 |  | MRSA |  | II |  | 1 |  | JADGTQ000000000 |
| IHMA78 |  | 2015 |  | Cornea |  | North America |  | I |  | ST8 |  | t008 |  | MRSA |  | IVa |  | 2 |  | JADGTP000000000 |
| IHMA79 |  | 2015 |  | Cornea |  | North America |  | III |  | ST30 |  | t7785 |  | MSSA |  | - |  | 4 |  | JADGTO000000000 |
| IHMA80 |  | 2015 |  | Cornea |  | North America |  | I |  | ST672 |  | t3841 |  | MSSA |  | - |  | 4 |  | JADPXG000000000 |
| IHMA81 |  | 2015 |  | Conjunctiva |  | North America |  | III |  | ST779 |  | t878 |  | MSSA |  | - |  | 2 |  | JADGTM000000000 |
| IHMA82 |  | 2015 |  | Eye |  | Europe |  | I |  | ST45 |  | t737 |  | MSSA |  | - |  | 2 |  | JADGTL000000000 |
| IHMA83 |  | 2015 |  | Cornea |  | North America |  | I |  | ST8 |  | t008 |  | MSSA |  | - |  | 4 |  | JADGTK000000000 |
| IHMA84 |  | 2015 |  | Eye |  | Europe |  | IV |  | ST121 |  | t645 |  | MSSA |  | - |  | 4 |  | JADGTJ000000000 |
| IHMA85 |  | 2015 |  | Cornea |  | North America |  | I |  | ST1750 |  | t008 |  | MRSA |  | IVa |  | 2 |  | JADGTI000000000 |
| IHMA86 |  | 2015 |  | Cornea |  | North America |  | I |  | ST1750 |  | t008 |  | MRSA |  | IVa |  | 2 |  | JADGTH000000000 |
| IHMA87 |  | 2016 |  | Eye |  | South America |  | I |  | ST97 |  | t10212 |  | MSSA |  | - |  | 2 |  | JADGTG000000000 |
| IHMA88 |  | 2016 |  | Eye |  | Europe |  | III |  | ST30 |  | t012 |  | MSSA |  | - |  | 4 |  | JADGTF000000000 |
| IHMA89 |  | 2016 |  | Cornea |  | North America |  | III |  | ST39 |  | t096 |  | MSSA |  | - |  | 3 |  | JADGTE000000000 |
| IHMA90 |  | 2016 |  | Cornea |  | North America |  | II |  | None |  | t1265 |  | MSSA |  | - |  | 1 |  | JADGTC000000000 |
| IHMA91 |  | 2016 |  | Cornea |  | North America |  | II |  | ST5 |  | t002 |  | MRSA |  | IVa |  | 1 |  | JADGTB000000000 |
| IHMA92 |  | 2016 |  | Cornea |  | North America |  | I |  | ST72 |  | t126 |  | MRSA |  | IVa |  | 1 |  | JADGTA000000000 |
| IHMA93 |  | 2016 |  | Cornea |  | North America |  | II |  | ST5 |  | t002 |  | MSSA |  | - |  | 1 |  | JADGSZ000000000 |
| IHMA94 |  | 2016 |  | Cornea |  | North America |  | I |  | ST361 |  | t315 |  | MSSA |  | - |  | 4 |  | JADGSY000000000 |
| IHMA95 |  | 2016 |  | Cornea |  | North America |  | II |  | ST5 |  | t242 |  | MRSA |  | II |  | 1 |  | JADGSX000000000 |
| IHMA96 |  | 2016 |  | Eye |  | Europe |  | IV |  | ST111 |  | Unknown |  | MRSA |  | I |  | 1 |  | JADGSW000000000 |
| IHMA97 |  | 2016 |  | Eye |  | Europe |  | I |  | ST22 |  | t1977 |  | MSSA |  | - |  | 4 |  | JADGSV000000000 |
| IHMA98 |  | 2016 |  | Eye |  | Asia |  | III |  | ST1 |  | t127 |  | MSSA |  | - |  | 3 |  | JADGSU000000000 |
| IHMA99 |  | 2016 |  | Eye |  | Europe |  | I |  | ST3975 |  | t091 |  | MSSA |  | - |  | 3 |  | JADGST000000000 |
| IHMA100 |  | 2016 |  | Eye |  | Europe |  | I |  | ST7 |  | t289 |  | MSSA |  | - |  | 3 |  | JADGYF000000000 |
| IHMA101 |  | 2016 |  | Cornea |  | North America |  | I |  | ST8 |  | t064 |  | MRSA |  | VIII |  | 2 |  | JADGYE000000000 |
| IHMA102 |  | 2016 |  | Cornea |  | North America |  | III |  | ST30 |  | t11456 |  | MSSA |  | - |  | 4 |  | JADGYD000000000 |
| IHMA103 |  | 2016 |  | Cornea |  | North America |  | I |  | ST7 |  | t796 |  | MSSA |  | - |  | 3 |  | JADGYC000000000 |
| IHMA104 |  | 2016 |  | Cornea |  | North America |  | I |  | ST8 |  | Unknown |  | MSSA |  | - |  | 2 |  | JADGYB000000000 |
| IHMA105 |  | 2016 |  | Cornea |  | North America |  | I |  | ST45 |  | Unknown |  | MSSA |  | - |  | 4 |  | JADGYA000000000 |
| IHMA106 |  | 2016 |  | Cornea |  | North America |  | II |  | None |  | t242 |  | MRSA |  | II |  | 1 |  | JADGXZ000000000 |
| IHMA107 |  | 2016 |  | Cornea |  | North America |  | I |  | ST45 |  | t015 |  | MSSA |  | - |  | 4 |  | JADGXY000000000 |
| IHMA108 |  | 2016 |  | Cornea |  | North America |  | III |  | ST88 |  | Unknown |  | MSSA |  | - |  | 3 |  | JADGXX000000000 |
| IHMA109 |  | 2016 |  | Cornea |  | North America |  | II |  | ST5 |  | t002 |  | MRSA |  | II |  | 1 |  | JADGXW000000000 |
| IHMA110 |  | 2015 |  | Eye |  | Europe |  | III |  | ST80 |  | t044 |  | MRSA |  | IVc |  | 3 |  | JADGXU000000000 |
| IHMA111 |  | 2016 |  | Eye |  | Europe |  | I |  | ST398 |  | t1255 |  | MSSA |  | - |  | 4 |  | JADGXT000000000 |
| IHMA112 |  | 2016 |  | Cornea |  | North America |  | II |  | ST5 |  | t105 |  | MRSA |  | II |  | 1 |  | JADGXS000000000 |
| IHMA113 |  | 2016 |  | Cornea |  | North America |  | II |  | ST5 |  | t002 |  | MRSA |  | II |  | 1 |  | JADGXR000000000 |
| IHMA114 |  | 2016 |  | Cornea |  | North America |  | II |  | ST109 |  | t209 |  | MSSA |  | - |  | 4 |  | JADGXQ000000000 |
| IHMA115 |  | 2016 |  | Cornea |  | North America |  | I |  | ST97 |  | t938 |  | MSSA |  | - |  | 2 |  | JADGXP000000000 |
| IHMA116 |  | 2016 |  | Cornea |  | North America |  | IV |  | ST51 |  | t159 |  | MSSA |  | - |  | 4 |  | JADGXO000000000 |
| IHMA117 |  | 2017 |  | Eye |  | Europe |  | II |  | ST582 |  | t084 |  | MSSA |  | - |  | 3 |  | JADGXN000000000 |
| IHMA118 |  | 2016 |  | Eye |  | North America |  | II |  | ST15 |  | t6684 |  | MSSA |  | - |  | 3 |  | JADGXM000000000 |
| IHMA119 |  | 2017 |  | Eye |  | Asia |  | I |  | ST8 |  | t008 |  | MSSA |  | - |  | 2 |  | JADGXL000000000 |
| IHMA120 |  | 2017 |  | Eye |  | Europe |  | II |  | None |  | Unknown |  | MRSA |  | II |  | 3 |  | JADGXJ000000000 |
| IHMA121 |  | 2017 |  | Eye |  | Europe |  | III |  | ST1930 |  | Unknown |  | MSSA |  | - |  | 3 |  | JADGXI000000000 |
| IHMA122 |  | 2017 |  | Eye |  | Europe |  | I |  | ST45 |  | t505 |  | MSSA |  | - |  | 4 |  | JADGXH000000000 |
| IHMA123 |  | 2017 |  | Eye |  | Europe |  | I |  | ST22 |  | t223 |  | MRSA |  | IVa |  | 4 |  | JADGXG000000000 |
| IHMA124 |  | 2016 |  | Eye |  | Europe |  | IV |  | ST121 |  | t645 |  | MSSA |  | - |  | 4 |  | JADGXF000000000 |
| IHMA125 |  | 2017 |  | Conjunctiva |  | North America |  | II |  | ST15 |  | t491 |  | MSSA |  | - |  | 3 |  | JADGXE000000000 |
| IHMA126 |  | 2017 |  | Conjunctiva |  | North America |  | I |  | ST8 |  | t008 |  | MRSA |  | IVa |  | 2 |  | JADGXD000000000 |
| IHMA127 |  | 2017 |  | Conjunctiva |  | North America |  | I |  | ST398 |  | Unknown |  | MSSA |  | - |  | 4 |  | JADGXC000000000 |
| IHMA128 |  | 2017 |  | Conjunctiva |  | North America |  | IV |  | ST59 |  | t7954 |  | MSSA |  | - |  | 3 |  | JADGXB000000000 |
| IHMA129 |  | 2017 |  | Conjunctiva |  | North America |  | III |  | ST81 |  | t127 |  | MSSA |  | - |  | 3 |  | JADGXA000000000 |
| IHMA130 |  | 2017 |  | Conjunctiva |  | North America |  | II |  | ST5 |  | t002 |  | MSSA |  | - |  | 1 |  | JADGWY000000000 |
| IHMA131 |  | 2017 |  | Conjunctiva |  | North America |  | III |  | ST207 |  | t525 |  | MSSA |  | - |  | 3 |  | JADGWX000000000 |
| IHMA132 |  | 2017 |  | Cornea |  | North America |  | IV |  | ST87 |  | t216 |  | MRSA |  | IVa |  | 3 |  | JADGWW000000000 |
| IHMA133 |  | 2017 |  | Conjunctiva |  | North America |  | I |  | ST8 |  | t051 |  | MSSA |  | - |  | 2 |  | JADGWV000000000 |
| IHMA134 |  | 2017 |  | Cornea |  | North America |  | I |  | ST8 |  | t008 |  | MSSA |  | - |  | 2 |  | JADGWU000000000 |
| IHMA135 |  | 2017 |  | Eye |  | Europe |  | I |  | ST45 |  | Unknown |  | MSSA |  | - |  | 4 |  | JADGWT000000000 |
| IHMA136 |  | 2017 |  | Eye |  | Africa |  | I |  | ST22 |  | Unknown |  | MRSA |  | IV |  | 4 |  | JADGWS000000000 |
| IHMA137 |  | 2017 |  | Eye |  | Europe |  | I |  | ST22 |  | t223 |  | MSSA |  | - |  | 4 |  | JADGWR000000000 |
| IHMA138 |  | 2017 |  | Conjunctiva |  | North America |  | II |  | ST15 |  | t084 |  | MSSA |  | - |  | 3 |  | JADGWQ000000000 |
| IHMA139 |  | 2017 |  | Eye |  | Europe |  | I |  | ST22 |  | t223 |  | MSSA |  | - |  | 4 |  | JADGWP000000000 |
| IHMA140 |  | 2017 |  | Eye |  | Africa |  | I |  | ST45 |  | t050 |  | MSSA |  | - |  | 4 |  | JADGWN000000000 |
| IHMA141 |  | 2017 |  | Conjunctiva |  | North America |  | I |  | ST6 |  | t304 |  | MSSA |  | - |  | 3 |  | JADGWM000000000 |
| IHMA142 |  | 2017 |  | Eye |  | South America |  | I |  | ST188 |  | t189 |  | MSSA |  | - |  | 3 |  | JADGWL000000000 |
| IHMA143 |  | 2017 |  | Conjunctiva |  | North America |  | II |  | ST5 |  | t242 |  | MSSA |  | - |  | 1 |  | JADGWK000000000 |
| IHMA144 |  | 2017 |  | Conjunctiva |  | North America |  | I |  | ST8 |  | t024 |  | MRSA |  | IVa |  | 2 |  | JADGWJ000000000 |
| IHMA145 |  | 2017 |  | Conjunctiva |  | North America |  | II |  | ST5 |  | t13280 |  | MSSA |  | - |  | 1 |  | JADGWI000000000 |
| IHMA146 |  | 2017 |  | Cornea |  | North America |  | I |  | ST72 |  | t148 |  | MSSA |  | - |  | 1 |  | JADGWH000000000 |
| IHMA147 |  | 2017 |  | Conjunctiva |  | North America |  | I |  | ST45 |  | t11984 |  | MSSA |  | - |  | 4 |  | JADGWG000000000 |
| IHMA148 |  | 2017 |  | Eye |  | Europe |  | III |  | ST30 |  | t021 |  | MSSA |  | - |  | 4 |  | JADGWF000000000 |
| IHMA149 |  | 2017 |  | Eye |  | Europe |  | III |  | ST30 |  | t122 |  | MSSA |  | - |  | 4 |  | JADGWE000000000 |
| URMC422 |  | 2019 |  | Cornea |  | North America |  | IV |  | ST51 |  | t272 |  | MSSA |  | - |  | 4 |  | JADGSS000000000 |
| URMC423 |  | 2019 |  | Cornea |  | North America |  | IV |  | ST59 |  | t216 |  | MRSA |  | II |  | 2 |  | JADGSR000000000 |
| URMC424 |  | 2019 |  | Cornea |  | North America |  | I |  | ST1181 |  | t334 |  | MSSA |  | - |  | 1 |  | JADGSQ000000000 |
| URMC425 |  | 2019 |  | Cornea |  | North America |  | I |  | ST72 |  | t148 |  | MSSA |  | - |  | 2 |  | JADGSP000000000 |
| URMC426 |  | 2019 |  | Cornea |  | North America |  | I |  | ST97 |  | t359 |  | MSSA |  | - |  | 3 |  | JADGSO000000000 |
| URMC427 |  | 2019 |  | Cornea |  | North America |  | IV |  | ST59 |  | t2871 |  | MRSA |  | II |  | 3 |  | JADGSN000000000 |
| URMC428 |  | 2019 |  | Cornea |  | North America |  | II |  | ST109 |  | t209 |  | MSSA |  | - |  | 4 |  | JADGSM000000000 |
| URMC429 |  | 2019 |  | Cornea |  | North America |  | II |  | ST5 |  | t002 |  | MRSA |  | II |  | 1 |  | JADGSL000000000 |
| URMC430 |  | 2019 |  | Cornea |  | North America |  | I |  | ST8 |  | t334 |  | MSSA |  | - |  | 2 |  | JADGSK000000000 |
| URMC431 |  | 2019 |  | Cornea |  | North America |  | III |  | ST30 |  | t012 |  | MSSA |  | - |  | 4 |  | JADGSJ000000000 |
| URMC432 |  | 2019 |  | Cornea |  | North America |  | I |  | ST72 |  | t148 |  | MSSA |  | - |  | 1 |  | JADGSI000000000 |
| URMC433 |  | 2019 |  | Cornea |  | North America |  | III |  | ST30 |  | t012 |  | MSSA |  | - |  | 4 |  | JADGSH000000000 |
| URMC434 |  | 2019 |  | Cornea |  | North America |  | II |  | ST5 |  | t002 |  | MSSA |  | - |  | 1 |  | JADGSG000000000 |
| URMC435 |  | 2019 |  | Cornea |  | North America |  | I |  | ST8 |  | t064 |  | MRSA |  | VIII |  | 2 |  | JADGSF000000000 |
