# Supplementary material for: Genomics of Staphylococcus aureus ocular isolates
Source: PLoS One. 2021 May 3;16(5):e0250975. doi: 10.1371/journal.pone.0250975 (PMC8092774; doi:10.1371/journal.pone.0250975)
Supplement: S2 Table — References are given for each isolate when available, with a dash indicating that no publicly available citation for the genome of an isolate could be identified. (DOCX) [file pone.0250975.s006.docx]

**S2 Table**. Source, date of isolation, geographic region, multilocus sequence type (MLST), methicillin resistance status, and SCC*mec* type of the 116 *Staphylococcus aureus* non-ocular isolates used in this study. References are given for each isolate when available, with a dash indicating that no publicly available citation for the genome of an isolate could be identified.

| Isolate |  | Date |  | Source |  | Region |  | MLST |  | MSSA/MRSA |  | SCC*mec* Type |  | Accession |  | Reference |
| --- | --- | --- | --- | --- | --- | --- | --- | --- | --- | --- | --- | --- | --- | --- | --- | --- |
| FORC_026 |  | 2014 |  | Blood |  | Asia |  | ST1 |  | MSSA |  | - |  | GCA_001879545.1 |  | [1] |
| ED98 |  | 1999 |  | Non-human |  | Europe |  | ST5 |  | MSSA |  | - |  | GCA_000024585.1 |  | [2] |
| FDAARGOS 159 |  | 2014 |  | Skin/Soft Tissue |  | North America |  | ST5 |  | MSSA |  | - |  | GCA_001558795.2 |  | [3] |
| FORC 027 |  | 2006 |  | Blood |  | Asia |  | ST5 |  | MRSA |  | II |  | GCA_001725965.1 |  | - |
| HOU1444-VR |  | 2012 |  | Blood |  | South America |  | ST5 |  | MSSA |  | - |  | GCA_001278745.1 |  | [4] |
| MI |  | 1997 |  | Heart |  | North America |  | ST5 |  | MRSA |  | II |  | GCA_001548415.1 |  | [5] |
| Mu3 |  | 1996 |  | Nasal |  | Asia |  | ST5 |  | MRSA |  | II |  | GCA_000010445.1 |  | [6] |
| Mu50 |  | 1997 |  | Skin/Soft Tissue |  | Asia |  | ST5 |  | MRSA |  | II |  | GCA_000009665.1 |  | [7] |
| N315 |  | 1982 |  | Nasal |  | Asia |  | ST5 |  | MRSA |  | II |  | GCA_000009645.1 |  | [8] |
| NCCP14558 |  | 2013 |  | Blood |  | Asia |  | ST5 |  | MRSA |  | II |  | GCA_001640905.1 |  | - |
| NCCP14562 |  | 2013 |  | Blood |  | Asia |  | ST5 |  | MRSA |  | II |  | GCA_001640925.1 |  | - |
| NZAK3 |  | 2005 |  | Skin |  | Asia |  | ST5 |  | MRSA |  | IV |  | GCA_900017775.1 |  | [9] |
| RIVM6519 |  | 2012 |  | Unknown |  | Europe |  | ST5 |  | MRSA |  | V |  | GCA_001618305.1 |  | [10] |
| SA564 |  | 2013 |  | Unknown |  | Europe |  | ST5 |  | MSSA |  | - |  | GCA_001281145.1 |  | [11] |
| UCI28 |  | 2009 |  | Unknown |  | North America |  | ST5 |  | MRSA |  | II |  | GCA_001975045.1 |  | [12] |
| UCI62 |  | 2010 |  | Unknown |  | North America |  | ST5 |  | MRSA |  | II |  | GCA_001975005.1 |  | [12] |
| ZJ5499 |  | 2010 |  | Skin/Soft Tissue |  | Asia |  | ST5 |  | MRSA |  | II |  | GCA_001640885.1 |  | - |
| 08-02300 |  | 2008 |  | Skin/Soft Tissue |  | Europe |  | ST7 |  | MSSA |  | - |  | GCA_001656075.1 |  | [13] |
| CA12 |  | 2007 |  | Blood |  | South America |  | ST8 |  | MRSA |  | IVc |  | GCA_001045795.2 |  | [14] |
| CA15 |  | 2007 |  | Blood |  | South America |  | ST8 |  | MRSA |  | IVa |  | GCA_001021895.1 |  | [14] |
| DSM 20231 |  | 1884 |  | Skin/Soft Tissue |  | Unknown |  | ST8 |  | MSSA |  | - |  | GCA_001027105.1 |  | [15] |
| HG001 |  | 2010 |  | Blood |  | Europe |  | ST8 |  | MSSA |  | - |  | GCA_001900185.1 |  | [16] |
| HUV05 |  | 2006 |  | Blood |  | South America |  | ST8 |  | MRSA |  | IVc |  | GCA_001045995.2 |  | [14] |
| M121 |  | 2004 |  | Nasal |  | South America |  | ST8 |  | MRSA |  | IVc |  | GCA_001021875.1 |  | [14] |
| NCTC 8325 |  | 2006 |  | Blood |  | North America |  | ST8 |  | MSSA |  | - |  | GCA_000013425.1 |  | [17] |
| PP HGAG QV30 2SC T4 |  | 1995 |  | Skin/Soft Tissue |  | Europe |  | ST8 |  | MRSA |  | IVa |  | GCA_900129335.1 |  | - |
| Saureu_1625_CO1 |  | 2011 |  | Skin/Soft Tissue |  | North America |  | ST8 |  | MRSA |  | IVa |  | GCA_001717725.2 |  | [18] |
| Saureu_1969_N |  | 2011 |  | Nose |  | North America |  | ST8 |  | MRSA |  | IVa |  | GCA_001717705.2 |  | [18] |
| Saureu_1971_CO1 |  | 2011 |  | Skin/Soft Tissue |  | North America |  | ST8 |  | MRSA |  | IVa |  | GCA_001717685.3 |  | [18] |
| Saureu_2148_CO1 |  | 2011 |  | Skin/Soft Tissue |  | North America |  | ST8 |  | MSSA |  | - |  | GCA_001717975.3 |  | [18] |
| Saureu_2395_USA500 |  | 1998 |  | Skin/Soft Tissue |  | North America |  | ST8 |  | MRSA |  | IVd |  | GCA_000746505.1 |  | [19] |
| TCH1516 |  | 2007 |  | Blood |  | North America |  | ST8 |  | MRSA |  | IVa |  | GCA_000017085.1 |  | [20] |
| UA_S391_USA300 |  | 2012 |  | Skin/Soft Tissue |  | Europe |  | ST8 |  | MRSA |  | IVa |  | GCA_000695875.1 |  | [21] |
| USA300_2014_C01 |  | 2011 |  | Skin/Soft Tissue |  | North America |  | ST8 |  | MRSA |  | IVa |  | GCA_001183705.3 |  | [22] |
| USA300_2014_C02 |  | 2011 |  | Skin/Soft Tissue |  | North America |  | ST8 |  | MRSA |  | IVa |  | GCA_001183725.2 |  | [22] |
| USA300 FPR3757 |  | 2006 |  | Joint |  | North America |  | ST8 |  | MRSA |  | IVa |  | GCA_000013465.1 |  | [23] |
| USA300_ISMMs1 |  | 2014 |  | Heart |  | North America |  | ST8 |  | MRSA |  | IVa |  | GCA_000568455.1 |  | [24] |
| USA300_SUR1 |  | 2013 |  | Perineum |  | South America |  | ST8 |  | MRSA |  | IVa |  | GCA_003595365.1 |  | [25] |
| USA300_SUR9 |  | 2013 |  | Nasal |  | South America |  | ST8 |  | MRSA |  | IVa |  | GCA_002000565.1 |  | [25] |
| USA300_SUR10 |  | 2013 |  | Nasal |  | South America |  | ST8 |  | MRSA |  | IVa |  | GCA_002000585.1 |  | [25] |
| USA300_SUR11 |  | 2013 |  | Perineum |  | South America |  | ST8 |  | MRSA |  | IVa |  | GCA_002000605.1 |  | [25] |
| USA300_SUR12 |  | 2013 |  | Skin/Soft Tissue |  | South America |  | ST8 |  | MRSA |  | IVa |  | GCA_002000625.1 |  | [25] |
| USA300_SUR13 |  | 2013 |  | Joint |  | South America |  | ST8 |  | MRSA |  | IVa |  | GCA_002000645.1 |  | [25] |
| USA300_SUR15 |  | 2013 |  | Nasal |  | South America |  | ST8 |  | MRSA |  | IVa |  | GCA_002000685.1 |  | [25] |
| USA300_SUR16 |  | 2013 |  | Perineum |  | South America |  | ST8 |  | MRSA |  | IVa |  | GCA_002000705.1 |  | [25] |
| USA300_SUR17 |  | 2013 |  | Nasal |  | South America |  | ST8 |  | MRSA |  | IVa |  | GCA_002000725.1 |  | [25] |
| USA300_SUR18 |  | 2013 |  | Nasal |  | South America |  | ST8 |  | MRSA |  | IVa |  | GCA_002000745.1 |  | [25] |
| USA300_SUR19 |  | 2013 |  | Perineum |  | South America |  | ST8 |  | MRSA |  | IVa |  | GCA_002000765.1 |  | [25] |
| USA300_SUR20 |  | 2013 |  | Nasal |  | South America |  | ST8 |  | MRSA |  | IVa |  | GCA_002000785.1 |  | [25] |
| USA300_SUR21 |  | 2013 |  | Skin/Soft Tissue |  | South America |  | ST8 |  | MRSA |  | IVa |  | GCA_002000805.1 |  | [25] |
| USA300_SUR22 |  | 2013 |  | Joint |  | South America |  | ST8 |  | MRSA |  | IVa |  | GCA_002000825.1 |  | [25] |
| USA300_SUR23 |  | 2013 |  | Perineum |  | South America |  | ST8 |  | MRSA |  | IVa |  | GCA_002000845.1 |  | [25] |
| USA300_SUR24 |  | 2013 |  | Perineum |  | South America |  | ST8 |  | MRSA |  | IVa |  | GCA_002000865.1 |  | [25] |
| UTSW_MRSA_55 |  | 2013 |  | Bone |  | North America |  | ST8 |  | MRSA |  | IVa |  | GCA_001580515.1 |  | [26] |
| VC40 |  | 2012 |  | Blood |  | Unknown |  | ST8 |  | MSSA |  | - |  | GCA_000245495.1 |  | [27] |
| ST20130939 |  | 2008 |  | Bone |  | Europe |  | ST15 |  | MSSA |  | - |  | GCA_001611425.1 |  | [28] |
| ST20130938 |  | 2009 |  | Bone |  | Europe |  | ST15 |  | MSSA |  | - |  | GCA_001611405.1 |  | [28] |
| ST20130940 |  | 2010 |  | Joint |  | Europe |  | ST15 |  | MSSA |  | - |  | GCA_001611325.1 |  | [28] |
| ST20130941 |  | 2010 |  | Joint |  | Europe |  | ST15 |  | MSSA |  | - |  | GCA_001611345.1 |  | [28] |
| HO50960412 |  | 2005 |  | Unknown |  | Europe |  | ST22 |  | MRSA |  | IV |  | GCA_000284535.1 |  | [29] |
| ST20130942 |  | 2009 |  | Joint |  | Europe |  | ST25 |  | MSSA |  | - |  | GCA_001611365.1 |  | [28] |
| ST20130943 |  | 2009 |  | Joint |  | Europe |  | ST25 |  | MSSA |  | - |  | GCA_001611385.1 |  | [28] |
| RKI4 |  | 2008 |  | Fecal Sample |  | Europe |  | ST27 |  | MSSA |  | - |  | GCA_001027045.1 |  | [30] |
| CA 347 |  | 2005 |  | Blood |  | North America |  | ST45 |  | MSSA |  | - |  | GCA_000412775.1 |  | [31] |
| MCRF184 |  | 2005 |  | Skin/Soft Tissue |  | North America |  | ST45 |  | MSSA |  | - |  | GCA_001594205.1 |  | [32] |
| Tager 104 |  | 1947 |  | Skin/Soft Tissue |  | North America |  | ST49 |  | MSSA |  | - |  | GCA_000452385.2 |  | [33] |
| Saureu 6850 |  | 1987 |  | Blood |  | North America |  | ST50 |  | MSSA |  | - |  | GCA_000462955.1 |  | [34] |
| M013 |  | 2002 |  | Skin/Soft Tissue |  | Asia |  | ST59 |  | MRSA |  | Vb |  | GCA_000237125.1 |  | [35] |
| SA40 |  | 2005 |  | Nasal |  | Asia |  | ST59 |  | MRSA |  | IVg |  | GCA_000470865.1 |  | [36] |
| SA40TW |  | 2005 |  | Nasal |  | Asia |  | ST59 |  | MRSA |  | IVg |  | GCA_001880265.1 |  | [36] |
| SA268 |  | 2012 |  | Blood |  | Asia |  | ST59 |  | MRSA |  | IV |  | GCA_000737615.1 |  | [37] |
| SA957 |  | 2000 |  | Blood |  | Asia |  | ST59 |  | MRSA |  | Vb |  | GCA_000470845.1 |  | [36] |
| 2148 N |  | 2011 |  | Nasal |  | North America |  | ST72 |  | MSSA |  | - |  | GCA_001717665.2 |  | [18] |
| CN1 |  | 2006 |  | Unknown |  | Asia |  | ST72 |  | MRSA |  | IVc |  | GCA_000463055.1 |  | [38] |
| FORC 012 |  | 2009 |  | Skin/Soft Tissue |  | Asia |  | ST72 |  | MRSA |  | IVb |  | GCA_001580495.1 |  | - |
| TMUS2126/TD1 |  | 2014 |  | Heart |  | Asia |  | ST72 |  | MRSA |  | IVc |  | GCA_001549655.1 |  | [39] |
| TMUS2134/TD4 |  | 2014 |  | Heart |  | Asia |  | ST72 |  | MRSA |  | IVc |  | GCA_001549675.1 |  | [39] |
| 11819-97 |  | 1997 |  | Skin/Soft Tissue |  | Europe |  | ST80 |  | MRSA |  | IVc |  | GCA_000239235.1 |  | [40] |
| GR2 |  | 2006 |  | Unknown |  | Europe |  | ST80 |  | MRSA |  | IVc |  | GCA_001296985.1 |  | [41] |
| SR434 |  | 2015 |  | Skin/Soft Tissue |  | Asia |  | ST88 |  | MRSA |  | IVc |  | GCA_001986135.1 |  | [42] |
| AUS0325 |  | 2013 |  | Joint |  | Asia |  | ST88 |  | MSSA |  | - |  | GCA_900096745.1 |  | [43] |
| JKD6159 |  | 2010 |  | Skin/Soft Tissue |  | Asia |  | ST93 |  | MRSA |  | IVa |  | GCA_000144955.1 |  | [44] |
| FCFHV36 |  | 2010 |  | Bone |  | South America |  | ST105 |  | MRSA |  | II |  | GCA_000969225.1 |  | [45] |
| JH1 |  | 2003 |  | Blood |  | North America |  | ST105 |  | MRSA |  | II |  | GCA_000017125.1 |  | [46] |
| JH9 |  | 2003 |  | Blood |  | North America |  | ST105 |  | MRSA |  | II |  | GCA_000016805.1 |  | [46] |
| XQ |  | 2009 |  | Skin/Soft Tissue |  | Asia |  | ST121 |  | MSSA |  | - |  | GCA_001444345.1 |  | [47] |
| ED133 |  | Unknown |  | Non-human |  | Europe |  | ST133 |  | MSSA |  | - |  | GCA_000210315.1 |  | [48] |
| BB155 |  | 2005 |  | Nasal |  | Africa |  | ST152 |  | MSSA |  | - |  | GCA_900004855.1 |  | - |
| NZ15MR0322 |  | 2015 |  | Skin/Soft Tissue |  | Asia |  | ST183 |  | MRSA |  | Vc |  | GCA_900155335.1 |  | - |
| Be62 |  | 1996 |  | Blood |  | South America |  | ST239 |  | MRSA |  | III |  | GCA_001515685.1 |  | [49] |
| Bmb9393 |  | 1993 |  | Blood |  | South America |  | ST239 |  | MRSA |  | III |  | GCA_000418345.1 |  | [50] |
| Gv51 |  | 1997 |  | Nasal |  | South America |  | ST239 |  | MRSA |  | III |  | GCA_001515665.1 |  | [51] |
| Gv69 |  | 1996 |  | Skin/Soft Tissue |  | South America |  | ST239 |  | MRSA |  | III |  | GCA_000769575.1 |  | [52] |
| Gv88 |  | 1997 |  | Skin/Soft Tissue |  | South America |  | ST239 |  | MRSA |  | III |  | GCA_001515705.1 |  | [51] |
| HC1335 |  | 2001 |  | Nasal |  | South America |  | ST239 |  | MRSA |  | III |  | GCA_001515765.1 |  | [49] |
| HC1340 |  | 2001 |  | Nasal |  | South America |  | ST239 |  | MRSA |  | III |  | GCA_001515745.1 |  | [49] |
| JKD6008 |  | 2003 |  | Blood |  | Asia |  | ST239 |  | MRSA |  | III |  | GCA_000145595.1 |  | [53] |
| T0131 |  | 2006 |  | Unknown |  | Asia |  | ST239 |  | MRSA |  | III |  | GCA_000204665.1 |  | [54] |
| TW20 |  | 2003 |  | Blood |  | Europe |  | ST239 |  | MRSA |  | III |  | GCA_000027045.1 |  | [29] |
| XN108 |  | 2004 |  | Skin/Soft Tissue |  | Asia |  | ST239 |  | MRSA |  | III |  | GCA_000709475.1 |  | [55] |
| Z172 |  | 2010 |  | Blood |  | Asia |  | ST239 |  | MRSA |  | III |  | GCA_000485885.1 |  | [56] |
| COL |  | 1960 |  | Operating Theater |  | Europe |  | ST250 |  | MRSA |  | I |  | GCA_000012045.1 |  | [57] |
| Newman |  | 1950 |  | Bone |  | Europe |  | ST254 |  | MSSA |  | - |  | GCA_000010465.1 |  | [58] |
| MS4 |  | 2012 |  | Bone |  | Asia |  | ST338 |  | MRSA |  | Vb |  | GCA_001456215.1 |  | [59] |
| 08S00974 |  | 2012 |  | Non-human |  | Europe |  | ST398 |  | MRSA |  | V |  | GCA_002025125.1 |  | [60] |
| 08BA02176 |  | 2008 |  | Skin/Soft Tissue |  | North America |  | ST398 |  | MRSA |  | V |  | GCA_000296595.1 |  | [61] |
| RIVM1295 |  | 2009 |  | Unknown |  | Europe |  | ST398 |  | MRSA |  | Vc |  | GCA_001465635.1 |  | [10] |
| RIVM1607 |  | 2008 |  | Unknown |  | Europe |  | ST398 |  | MRSA |  | Vc |  | GCA_001465675.1 |  | [10] |
| RIVM3897 |  | 2008 |  | Unknown |  | Europe |  | ST398 |  | MRSA |  | V |  | GCA_001465755.1 |  | [10] |
| SJTUF_J27 |  | 2007 |  | Non-human |  | Asia |  | ST433 |  | MSSA |  | - |  | GCF_001956755.1 |  | [62] |
| ATCC 6538 |  | 1884 |  | Skin/Soft Tissue |  | Europe |  | ST464 |  | MSSA |  | - |  | GCA_002025145.1 |  | [63] |
| FDA209P |  | 2015 |  | Laboratory Strain |  | North America |  | ST464 |  | MSSA |  | - |  | GCA_001548295.1 |  | [64] |
| 08-02119 |  | 2008 |  | Skin/Soft Tissue |  | Europe |  | ST582 |  | MSSA |  | - |  | GCA_001656045.1 |  | [13] |
| DAR4145 |  | 2009 |  | Blood |  | Asia |  | ST772 |  | MRSA |  | V |  | GCA_000828035.1 |  | [65] |
| V2200 |  | 2007 |  | Bone |  | South America |  | ST923 |  | MRSA |  | IVa |  | GCA_001046095.2 |  | [14] |
| JS395 |  | 1998 |  | Unknown |  | Europe |  | ST1093 |  | MRSA |  | IVg |  | GCA_001307235.1 |  | [66] |

1. Ko DS, Seong WJ, Kim D, Kim EK, Kim NH, Lee CY, et al. Molecular prophage typing of Staphylococcus aureus isolates from bovine mastitis. J Vet Sci. 2018;19(6):771-81. Epub 2018/09/04. doi: 10.4142/jvs.2018.19.6.771. PubMed PMID: 30173494; PubMed Central PMCID: PMCPMC6265582.

2. Lowder BV, Guinane CM, Ben Zakour NL, Weinert LA, Conway-Morris A, Cartwright RA, et al. Recent human-to-poultry host jump, adaptation, and pandemic spread of Staphylococcus aureus. Proc Natl Acad Sci U S A. 2009;106(46):19545-50. Epub 2009/11/04. doi: 10.1073/pnas.0909285106. PubMed PMID: 19884497; PubMed Central PMCID: PMCPMC2780746.

3. Sichtig H, Minogue T, Yan Y, Stefan C, Hall A, Tallon L, et al. FDA-ARGOS is a database with public quality-controlled reference genomes for diagnostic use and regulatory science. Nat Commun. 2019;10(1):3313. Epub 2019/07/28. doi: 10.1038/s41467-019-11306-6. PubMed PMID: 31346170; PubMed Central PMCID: PMCPMC6658474.

4. Panesso D, Planet PJ, Diaz L, Hugonnet JE, Tran TT, Narechania A, et al. Methicillin-Susceptible, Vancomycin-Resistant Staphylococcus aureus, Brazil. Emerg Infect Dis. 2015;21(10):1844-8. Epub 2015/09/25. doi: 10.3201/eid2110.141914. PubMed PMID: 26402569; PubMed Central PMCID: PMCPMC4593430.

5. Hishinuma T, Katayama Y, Matsuo M, Sasaki T, Hiramatsu K. Complete Genome Sequence of Vancomycin-Intermediate Staphylococcus aureus Strain MI (HIP5827). Genome Announc. 2016;4(2). Epub 2016/03/19. doi: 10.1128/genomeA.00123-16. PubMed PMID: 26988042; PubMed Central PMCID: PMCPMC4796121.

6. Hiramatsu K, Aritaka N, Hanaki H, Kawasaki S, Hosoda Y, Hori S, et al. Dissemination in Japanese hospitals of strains of Staphylococcus aureus heterogeneously resistant to vancomycin. Lancet. 1997;350(9092):1670-3. Epub 1997/12/24. doi: 10.1016/S0140-6736(97)07324-8. PubMed PMID: 9400512.

7. Ohta T, Hirakawa H, Morikawa K, Maruyama A, Inose Y, Yamashita A, et al. Nucleotide substitutions in Staphylococcus aureus strains, Mu50, Mu3, and N315. DNA Res. 2004;11(1):51-6. Epub 2004/05/15. doi: 10.1093/dnares/11.1.51. PubMed PMID: 15141945.

8. Kuroda M, Ohta T, Uchiyama I, Baba T, Yuzawa H, Kobayashi I, et al. Whole genome sequencing of meticillin-resistant Staphylococcus aureus. Lancet. 2001;357(9264):1225-40. Epub 2001/06/22. doi: 10.1016/s0140-6736(00)04403-2. PubMed PMID: 11418146.

9. Baines SL, Howden BP, Heffernan H, Stinear TP, Carter GP, Seemann T, et al. Rapid Emergence and Evolution of Staphylococcus aureus Clones Harboring fusC-Containing Staphylococcal Cassette Chromosome Elements. Antimicrob Agents Chemother. 2016;60(4):2359-65. Epub 2016/02/10. doi: 10.1128/AAC.03020-15. PubMed PMID: 26856837; PubMed Central PMCID: PMCPMC4808225.

10. Bosch T, Witteveen S, Haenen A, Landman F, Schouls LM. Next-Generation Sequencing Confirms Presumed Nosocomial Transmission of Livestock-Associated Methicillin-Resistant Staphylococcus aureus in the Netherlands. Appl Environ Microbiol. 2016;82(14):4081-9. Epub 2016/05/01. doi: 10.1128/AEM.00773-16. PubMed PMID: 27129960; PubMed Central PMCID: PMCPMC4959200.

11. Giraud C, Hausmann S, Lemeille S, Prados J, Redder P, Linder P. The C-terminal region of the RNA helicase CshA is required for the interaction with the degradosome and turnover of bulk RNA in the opportunistic pathogen Staphylococcus aureus. RNA Biol. 2015;12(6):658-74. Epub 2015/05/23. doi: 10.1080/15476286.2015.1035505. PubMed PMID: 25997461; PubMed Central PMCID: PMCPMC4615653.

12. Hau SJ, Bayles DO, Alt DP, Nicholson TL. Complete Genome Sequences of Two Staphylococcus aureus Sequence Type 5 Isolates from California, USA. Genome Announc. 2017;5(13). Epub 2017/04/01. doi: 10.1128/genomeA.00099-17. PubMed PMID: 28360167; PubMed Central PMCID: PMCPMC5374241.

13. Weber RE, Layer F, Fuchs S, Bender JK, Fiedler S, Werner G, et al. Complete Genome Sequences of Two Methicillin-Sensitive Staphylococcus aureus Isolates Representing a Population Subset Highly Prevalent in Human Colonization. Genome Announc. 2016;4(4). Epub 2016/07/30. doi: 10.1128/genomeA.00716-16. PubMed PMID: 27469954; PubMed Central PMCID: PMCPMC4966458.

14. Planet PJ, Diaz L, Kolokotronis SO, Narechania A, Reyes J, Xing G, et al. Parallel Epidemics of Community-Associated Methicillin-Resistant Staphylococcus aureus USA300 Infection in North and South America. J Infect Dis. 2015;212(12):1874-82. Epub 2015/06/07. doi: 10.1093/infdis/jiv320. PubMed PMID: 26048971; PubMed Central PMCID: PMCPMC4655856.

15. Shiroma A, Terabayashi Y, Nakano K, Shimoji M, Tamotsu H, Ashimine N, et al. First Complete Genome Sequences of Staphylococcus aureus subsp. aureus Rosenbach 1884 (DSM 20231T), Determined by PacBio Single-Molecule Real-Time Technology. Genome Announc. 2015;3(4). Epub 2015/07/18. doi: 10.1128/genomeA.00800-15. PubMed PMID: 26184947; PubMed Central PMCID: PMCPMC4505135.

16. Caldelari I, Chane-Woon-Ming B, Noirot C, Moreau K, Romby P, Gaspin C, et al. Complete Genome Sequence and Annotation of the Staphylococcus aureus Strain HG001. Genome Announc. 2017;5(32). Epub 2017/08/12. doi: 10.1128/genomeA.00783-17. PubMed PMID: 28798184; PubMed Central PMCID: PMCPMC5552993.

17. Berscheid A, Sass P, Weber-Lassalle K, Cheung AL, Bierbaum G. Revisiting the genomes of the Staphylococcus aureus strains NCTC 8325 and RN4220. Int J Med Microbiol. 2012;302(2):84-7. Epub 2012/03/16. doi: 10.1016/j.ijmm.2012.01.002. PubMed PMID: 22417616.

18. LaBreck PT, Rice GK, Paskey AC, Elassal EM, Cer RZ, Law NN, et al. Conjugative Transfer of a Novel Staphylococcal Plasmid Encoding the Biocide Resistance Gene, qacA. Front Microbiol. 2018;9:2664. Epub 2018/12/05. doi: 10.3389/fmicb.2018.02664. PubMed PMID: 30510541; PubMed Central PMCID: PMCPMC6252503.

19. Benson MA, Ohneck EA, Ryan C, Alonzo F, 3rd, Smith H, Narechania A, et al. Evolution of hypervirulence by a MRSA clone through acquisition of a transposable element. Mol Microbiol. 2014;93(4):664-81. Epub 2014/06/26. doi: 10.1111/mmi.12682. PubMed PMID: 24962815; PubMed Central PMCID: PMCPMC4127135.

20. Highlander SK, Hulten KG, Qin X, Jiang H, Yerrapragada S, Mason EO, Jr., et al. Subtle genetic changes enhance virulence of methicillin resistant and sensitive Staphylococcus aureus. BMC Microbiol. 2007;7:99. Epub 2007/11/08. doi: 10.1186/1471-2180-7-99. PubMed PMID: 17986343; PubMed Central PMCID: PMCPMC2222628.

21. Sabirova JS, Xavier BB, Hernalsteens JP, De Greve H, Ieven M, Goossens H, et al. Complete Genome Sequences of Two Prolific Biofilm-Forming Staphylococcus aureus Isolates Belonging to USA300 and EMRSA-15 Clonal Lineages. Genome Announc. 2014;2(3). Epub 2014/06/28. doi: 10.1128/genomeA.00610-14. PubMed PMID: 24970829; PubMed Central PMCID: PMCPMC4073113.

22. Johnson RC, Schlett CD, Crawford K, Lanier JB, Merrell DS, Ellis MW. Recurrent Methicillin-Resistant Staphylococcus aureus Cutaneous Abscesses and Selection of Reduced Chlorhexidine Susceptibility during Chlorhexidine Use. J Clin Microbiol. 2015;53(11):3677-82. Epub 2015/08/21. doi: 10.1128/JCM.01771-15. PubMed PMID: 26292295; PubMed Central PMCID: PMCPMC4609685.

23. Diep BA, Gill SR, Chang RF, Phan TH, Chen JH, Davidson MG, et al. Complete genome sequence of USA300, an epidemic clone of community-acquired meticillin-resistant Staphylococcus aureus. Lancet. 2006;367(9512):731-9. Epub 2006/03/07. doi: 10.1016/S0140-6736(06)68231-7. PubMed PMID: 16517273.

24. Altman DR, Sebra R, Hand J, Attie O, Deikus G, Carpini KW, et al. Transmission of methicillin-resistant Staphylococcus aureus via deceased donor liver transplantation confirmed by whole genome sequencing. Am J Transplant. 2014;14(11):2640-4. Epub 2014/09/25. doi: 10.1111/ajt.12897. PubMed PMID: 25250641; PubMed Central PMCID: PMCPMC4651443.

25. Sabat AJ, Hermelijn SM, Akkerboom V, Juliana A, Degener JE, Grundmann H, et al. Complete-genome sequencing elucidates outbreak dynamics of CA-MRSA USA300 (ST8-spa t008) in an academic hospital of Paramaribo, Republic of Suriname. Sci Rep. 2017;7:41050. Epub 2017/01/21. doi: 10.1038/srep41050. PubMed PMID: 28106148; PubMed Central PMCID: PMCPMC5247709.

26. Otarigho B, Falade MO. Analysis of antibiotics resistant genes in different strains of Staphylococcus aureus. Bioinformation. 2018;14(3):113-22. Epub 2018/05/23. doi: 10.6026/97320630014113. PubMed PMID: 29785070; PubMed Central PMCID: PMCPMC5953858.

27. Sass P, Berscheid A, Jansen A, Oedenkoven M, Szekat C, Strittmatter A, et al. Genome sequence of Staphylococcus aureus VC40, a vancomycin- and daptomycin-resistant strain, to study the genetics of development of resistance to currently applied last-resort antibiotics. J Bacteriol. 2012;194(8):2107-8. Epub 2012/03/31. doi: 10.1128/JB.06631-11. PubMed PMID: 22461548; PubMed Central PMCID: PMCPMC3318483.

28. Trouillet-Assant S, Lelievre L, Martins-Simoes P, Gonzaga L, Tasse J, Valour F, et al. Adaptive processes of Staphylococcus aureus isolates during the progression from acute to chronic bone and joint infections in patients. Cell Microbiol. 2016;18(10):1405-14. Epub 2016/02/27. doi: 10.1111/cmi.12582. PubMed PMID: 26918656.

29. Holden MT, Lindsay JA, Corton C, Quail MA, Cockfield JD, Pathak S, et al. Genome sequence of a recently emerged, highly transmissible, multi-antibiotic- and antiseptic-resistant variant of methicillin-resistant Staphylococcus aureus, sequence type 239 (TW). J Bacteriol. 2010;192(3):888-92. Epub 2009/12/02. doi: 10.1128/JB.01255-09. PubMed PMID: 19948800; PubMed Central PMCID: PMCPMC2812470.

30. Stevens MJ, Stephan R, Johler S. Complete and Assembled Genome Sequence of Staphylococcus aureus RKI4, a Food-Poisoning Strain Exhibiting a Novel S. aureus Pathogenicity Island Carrying seb. Genome Announc. 2015;3(4). Epub 2015/07/04. doi: 10.1128/genomeA.00769-15. PubMed PMID: 26139727; PubMed Central PMCID: PMCPMC4490855.

31. Stegger M, Driebe EM, Roe C, Lemmer D, Bowers JR, Engelthaler DM, et al. Genome Sequence of Staphylococcus aureus Strain CA-347, a USA600 Methicillin-Resistant Isolate. Genome Announc. 2013;1(4). Epub 2013/07/28. doi: 10.1128/genomeA.00517-13. PubMed PMID: 23887918; PubMed Central PMCID: PMCPMC3735070.

32. Aswani V, Mau B, Shukla SK. Complete Genome Sequence of Staphylococcus aureus MCRF184, a Necrotizing Fasciitis-Causing Methicillin-Sensitive Sequence Type 45 Staphylococcus Strain. Genome Announc. 2016;4(3). Epub 2016/05/14. doi: 10.1128/genomeA.00374-16. PubMed PMID: 27174283; PubMed Central PMCID: PMCPMC4866860.

33. Davis RWt, Brannen AD, Hossain MJ, Monsma S, Bock PE, Nahrendorf M, et al. Complete genome of Staphylococcus aureus Tager 104 provides evidence of its relation to modern systemic hospital-acquired strains. BMC Genomics. 2016;17:179. Epub 2016/03/05. doi: 10.1186/s12864-016-2433-8. PubMed PMID: 26940863; PubMed Central PMCID: PMCPMC4778325.

34. Fraunholz M, Bernhardt J, Schuldes J, Daniel R, Hecker M, Sinha B. Complete Genome Sequence of Staphylococcus aureus 6850, a Highly Cytotoxic and Clinically Virulent Methicillin-Sensitive Strain with Distant Relatedness to Prototype Strains. Genome Announc. 2013;1(5). Epub 2013/09/28. doi: 10.1128/genomeA.00775-13. PubMed PMID: 24072870; PubMed Central PMCID: PMCPMC3784790.

35. Huang TW, Chen FJ, Miu WC, Liao TL, Lin AC, Huang IW, et al. Complete genome sequence of Staphylococcus aureus M013, a pvl-positive, ST59-SCCmec type V strain isolated in Taiwan. J Bacteriol. 2012;194(5):1256-7. Epub 2012/02/14. doi: 10.1128/JB.06666-11. PubMed PMID: 22328755; PubMed Central PMCID: PMCPMC3294775.

36. Chen CJ, Unger C, Hoffmann W, Lindsay JA, Huang YC, Gotz F. Characterization and comparison of 2 distinct epidemic community-associated methicillin-resistant Staphylococcus aureus clones of ST59 lineage. PLoS One. 2013;8(9):e63210. Epub 2013/09/17. doi: 10.1371/journal.pone.0063210. PubMed PMID: 24039691; PubMed Central PMCID: PMCPMC3764004.

37. Qu T, Feng Y, Jiang Y, Zhu P, Wei Z, Chen Y, et al. Whole genome analysis of a community-associated methicillin-resistant Staphylococcus aureus ST59 isolate from a case of human sepsis and severe pneumonia in China. PLoS One. 2014;9(2):e89235. Epub 2014/03/04. doi: 10.1371/journal.pone.0089235. PubMed PMID: 24586619; PubMed Central PMCID: PMCPMC3930696.

38. Chen Y, Chatterjee SS, Porcella SF, Yu YS, Otto M. Complete genome sequence of a Panton-Valentine leukocidin-negative community-associated methicillin-resistant Staphylococcus aureus strain of sequence type 72 from Korea. PLoS One. 2013;8(8):e72803. Epub 2013/08/27. doi: 10.1371/journal.pone.0072803. PubMed PMID: 23977354; PubMed Central PMCID: PMCPMC3748019.

39. Yamaguchi T, Suzuki S, Okamura S, Miura Y, Tsukimori A, Nakamura I, et al. Evolution and single-nucleotide polymorphisms in methicillin-resistant Staphylococcus aureus strains with reduced susceptibility to vancomycin and daptomycin, based on determination of the complete genome. Antimicrob Agents Chemother. 2015;59(6):3585-7. Epub 2015/03/18. doi: 10.1128/AAC.05159-14. PubMed PMID: 25779584; PubMed Central PMCID: PMCPMC4432144.

40. Stegger M, Price LB, Larsen AR, Gillece JD, Waters AE, Skov R, et al. Genome sequence of Staphylococcus aureus strain 11819-97, an ST80-IV European community-acquired methicillin-resistant isolate. J Bacteriol. 2012;194(6):1625-6. Epub 2012/03/01. doi: 10.1128/JB.06653-11. PubMed PMID: 22374956; PubMed Central PMCID: PMCPMC3294860.

41. Sabat AJ, Pournaras S, Akkerboom V, Tsakris A, Grundmann H, Friedrich AW. Whole-genome analysis of an oxacillin-susceptible CC80 mecA-positive Staphylococcus aureus clinical isolate: insights into the mechanisms of cryptic methicillin resistance. J Antimicrob Chemother. 2015;70(11):2956-64. Epub 2015/07/23. doi: 10.1093/jac/dkv210. PubMed PMID: 26198147.

42. Sun L, Wu D, Chen Y, Wang Q, Wang H, Yu Y. Characterization of a PVL-negative community-acquired methicillin-resistant Staphylococcus aureus strain of sequence type 88 in China. Int J Med Microbiol. 2017;307(6):346-52. Epub 2017/07/25. doi: 10.1016/j.ijmm.2017.07.002. PubMed PMID: 28734577.

43. Kpeli G, Buultjens AH, Giulieri S, Owusu-Mireku E, Aboagye SY, Baines SL, et al. Genomic analysis of ST88 community-acquired methicillin resistant Staphylococcus aureus in Ghana. PeerJ. 2017;5:e3047. Epub 2017/03/08. doi: 10.7717/peerj.3047. PubMed PMID: 28265515; PubMed Central PMCID: PMCPMC5333547.

44. Chua K, Seemann T, Harrison PF, Davies JK, Coutts SJ, Chen H, et al. Complete genome sequence of Staphylococcus aureus strain JKD6159, a unique Australian clone of ST93-IV community methicillin-resistant Staphylococcus aureus. J Bacteriol. 2010;192(20):5556-7. Epub 2010/08/24. doi: 10.1128/JB.00878-10. PubMed PMID: 20729356; PubMed Central PMCID: PMCPMC2950503.

45. McCulloch JA, Silveira AC, Lima Moraes Ada C, Perez-Chaparro PJ, Ferreira Silva M, Almeida LM, et al. Complete Genome Sequence of Staphylococcus aureus FCFHV36, a Methicillin-Resistant Strain Heterogeneously Resistant to Vancomycin. Genome Announc. 2015;3(4). Epub 2015/08/15. doi: 10.1128/genomeA.00893-15. PubMed PMID: 26272570; PubMed Central PMCID: PMCPMC4536681.

46. Mwangi MM, Wu SW, Zhou Y, Sieradzki K, de Lencastre H, Richardson P, et al. Tracking the in vivo evolution of multidrug resistance in Staphylococcus aureus by whole-genome sequencing. Proc Natl Acad Sci U S A. 2007;104(22):9451-6. Epub 2007/05/23. doi: 10.1073/pnas.0609839104. PubMed PMID: 17517606; PubMed Central PMCID: PMCPMC1890515.

47. Zheng Y, Shang W, Peng H, Rao Y, Zhao X, Hu Z, et al. Virulence Determinants Are Required for Brain Abscess Formation Through Staphylococcus aureus Infection and Are Potential Targets of Antivirulence Factor Therapy. Front Microbiol. 2019;10:682. Epub 2019/04/27. doi: 10.3389/fmicb.2019.00682. PubMed PMID: 31024479; PubMed Central PMCID: PMCPMC6460967.

48. Guinane CM, Ben Zakour NL, Tormo-Mas MA, Weinert LA, Lowder BV, Cartwright RA, et al. Evolutionary genomics of Staphylococcus aureus reveals insights into the origin and molecular basis of ruminant host adaptation. Genome Biol Evol. 2010;2:454-66. Epub 2010/07/14. doi: 10.1093/gbe/evq031. PubMed PMID: 20624747; PubMed Central PMCID: PMCPMC2997551.

49. Botelho AMN, Cerqueira ECMO, Moustafa AM, Beltrame CO, Ferreira FA, Cortes MF, et al. Local Diversification of Methicillin- Resistant Staphylococcus aureus ST239 in South America After Its Rapid Worldwide Dissemination. Front Microbiol. 2019;10:82. Epub 2019/03/16. doi: 10.3389/fmicb.2019.00082. PubMed PMID: 30873127; PubMed Central PMCID: PMCPMC6400870.

50. Costa MO, Beltrame CO, Ferreira FA, Botelho AM, Lima NC, Souza RC, et al. Complete Genome Sequence of a Variant of the Methicillin-Resistant Staphylococcus aureus ST239 Lineage, Strain BMB9393, Displaying Superior Ability To Accumulate ica-Independent Biofilm. Genome Announc. 2013;1(4). Epub 2013/08/10. doi: 10.1128/genomeA.00576-13. PubMed PMID: 23929475; PubMed Central PMCID: PMCPMC3738891.

51. Aparecida Guimaraes M, Rocchetto Coelho L, Rodrigues Souza R, Ferreira-Carvalho BT, Marie Sa Figueiredo A. Impact of biocides on biofilm formation by methicillin-resistant Staphylococcus aureus (ST239-SCCmecIII) isolates. Microbiol Immunol. 2012;56(3):203-7. Epub 2012/01/04. doi: 10.1111/j.1348-0421.2011.00423.x. PubMed PMID: 22211887.

52. Botelho AMN, Costa MOC, Beltrame CO, Ferreira FA, Cortes MF, Bandeira PT, et al. Complete genome sequence of an agr-dysfunctional variant of the ST239 lineage of the methicillin-resistant Staphylococcus aureus strain GV69 from Brazil. Stand Genomic Sci. 2016;11:34. Epub 2016/05/07. doi: 10.1186/s40793-016-0154-x. PubMed PMID: 27152133; PubMed Central PMCID: PMCPMC4857242.

53. Howden BP, Seemann T, Harrison PF, McEvoy CR, Stanton JA, Rand CJ, et al. Complete genome sequence of Staphylococcus aureus strain JKD6008, an ST239 clone of methicillin-resistant Staphylococcus aureus with intermediate-level vancomycin resistance. J Bacteriol. 2010;192(21):5848-9. Epub 2010/08/31. doi: 10.1128/JB.00951-10. PubMed PMID: 20802046; PubMed Central PMCID: PMCPMC2953705.

54. Li Y, Cao B, Zhang Y, Zhou J, Yang B, Wang L. Complete genome sequence of Staphylococcus aureus T0131, an ST239-MRSA-SCCmec type III clone isolated in China. J Bacteriol. 2011;193(13):3411-2. Epub 2011/05/10. doi: 10.1128/JB.05135-11. PubMed PMID: 21551295; PubMed Central PMCID: PMCPMC3133253.

55. Zhang X, Xu X, Yuan W, Hu Q, Shang W, Hu X, et al. Complete Genome Sequence of Staphylococcus aureus XN108, an ST239-MRSA-SCCmec III Strain with Intermediate Vancomycin Resistance Isolated in Mainland China. Genome Announc. 2014;2(4). Epub 2014/07/26. doi: 10.1128/genomeA.00449-14. PubMed PMID: 25059856; PubMed Central PMCID: PMCPMC4110214.

56. Chen FJ, Lauderdale TL, Wang LS, Huang IW. Complete Genome Sequence of Staphylococcus aureus Z172, a Vancomycin-Intermediate and Daptomycin-Nonsusceptible Methicillin-Resistant Strain Isolated in Taiwan. Genome Announc. 2013;1(6). Epub 2013/12/07. doi: 10.1128/genomeA.01011-13. PubMed PMID: 24309740; PubMed Central PMCID: PMCPMC3853063.

57. Gill SR, Fouts DE, Archer GL, Mongodin EF, Deboy RT, Ravel J, et al. Insights on evolution of virulence and resistance from the complete genome analysis of an early methicillin-resistant Staphylococcus aureus strain and a biofilm-producing methicillin-resistant Staphylococcus epidermidis strain. J Bacteriol. 2005;187(7):2426-38. Epub 2005/03/19. doi: 10.1128/JB.187.7.2426-2438.2005. PubMed PMID: 15774886; PubMed Central PMCID: PMCPMC1065214.

58. Baba T, Bae T, Schneewind O, Takeuchi F, Hiramatsu K. Genome sequence of Staphylococcus aureus strain Newman and comparative analysis of staphylococcal genomes: polymorphism and evolution of two major pathogenicity islands. J Bacteriol. 2008;190(1):300-10. Epub 2007/10/24. doi: 10.1128/JB.01000-07. PubMed PMID: 17951380; PubMed Central PMCID: PMCPMC2223734.

59. Bai B, Lin Z, Pu Z, Xu G, Zhang F, Chen Z, et al. In vitro Activity and Heteroresistance of Omadacycline Against Clinical Staphylococcus aureus Isolates From China Reveal the Impact of Omadacycline Susceptibility by Branched-Chain Amino Acid Transport System II Carrier Protein, Na/Pi Cotransporter Family Protein, and Fibronectin-Binding Protein. Front Microbiol. 2019;10:2546. Epub 2019/12/04. doi: 10.3389/fmicb.2019.02546. PubMed PMID: 31787948; PubMed Central PMCID: PMCPMC6856048.

60. Makarova O, Johnston P, Walther B, Rolff J, Roesler U. Complete Genome Sequence of the Livestock-Associated Methicillin-Resistant Strain Staphylococcus aureus subsp. aureus 08S00974 (Sequence Type 398). Genome Announc. 2017;5(19). Epub 2017/05/13. doi: 10.1128/genomeA.00294-17. PubMed PMID: 28495772; PubMed Central PMCID: PMCPMC5427207.

61. Golding GR, Bryden L, Levett PN, McDonald RR, Wong A, Graham MR, et al. whole-genome sequence of livestock-associated st398 methicillin-resistant staphylococcus aureus Isolated from Humans in Canada. J Bacteriol. 2012;194(23):6627-8. Epub 2012/11/13. doi: 10.1128/JB.01680-12. PubMed PMID: 23144384; PubMed Central PMCID: PMCPMC3497547.

62. Xie Y, He Y, Ghatak S, Irwin P, Yan X, Strobaugh TP, Jr., et al. Whole-genome sequence data and analysis of a Staphylococcus aureus strain SJTUF_J27 isolated from seaweed. Data Brief. 2018;20:894-8. Epub 2018/09/19. doi: 10.1016/j.dib.2018.08.084. PubMed PMID: 30225298; PubMed Central PMCID: PMCPMC6138985.

63. Makarova O, Johnston P, Walther B, Rolff J, Roesler U. Complete Genome Sequence of the Disinfectant Susceptibility Testing Reference Strain Staphylococcus aureus subsp. aureus ATCC 6538. Genome Announc. 2017;5(19). Epub 2017/05/13. doi: 10.1128/genomeA.00293-17. PubMed PMID: 28495771; PubMed Central PMCID: PMCPMC5427206.

64. Singh M, Sasaki T, Matsuo M, Morimoto Y, Aiba Y, Hiramatsu K. Complete Genome Sequence of the Drug-Naive Classical Staphylococcus aureus Strain FDA209P. Genome Announc. 2015;3(6). Epub 2015/11/14. doi: 10.1128/genomeA.01343-15. PubMed PMID: 26564052; PubMed Central PMCID: PMCPMC4972786.

65. Steinig EJ, Andersson P, Harris SR, Sarovich DS, Manoharan A, Coupland P, et al. Single-molecule sequencing reveals the molecular basis of multidrug-resistance in ST772 methicillin-resistant Staphylococcus aureus. BMC Genomics. 2015;16:388. Epub 2015/05/20. doi: 10.1186/s12864-015-1599-9. PubMed PMID: 25981586; PubMed Central PMCID: PMCPMC4432960.

66. Winstel V, Liang C, Sanchez-Carballo P, Steglich M, Munar M, Broker BM, et al. Wall teichoic acid structure governs horizontal gene transfer between major bacterial pathogens. Nat Commun. 2013;4:2345. Epub 2013/08/24. doi: 10.1038/ncomms3345. PubMed PMID: 23965785; PubMed Central PMCID: PMCPMC3903184.
