# Supplementary material for: Genomics of Staphylococcus aureus ocular isolates
Source: PLoS One. 2021 May 3;16(5):e0250975. doi: 10.1371/journal.pone.0250975 (PMC8092774; doi:10.1371/journal.pone.0250975)
Supplement: S3 Table — (DOCX) [file pone.0250975.s007.docx]

**S3 Table.** Class, gene identity, function, and reference genome of the 235 *Staphylococcus aureus* virulence factor database.

| Functional Class |  | *Gene* |  | Description |  | *S. aureus* reference genome |
| --- | --- | --- | --- | --- | --- | --- |
| Adherence |  | *clfA* |  | Clumping factor A, fibrinogen binding protein |  | MW2 |
|  |  | *clfB* |  | Clumping factor B, fibrinogen binding protein |  | MW2 |
|  |  | *cna* |  | Collagen adhesion precursor |  | MW2 |
|  |  | *coa* |  | Stapylocoagulase precursor |  | N315 |
|  |  | *eap/map* |  | Truncated map-W protein |  | Mu50 |
|  |  | *ebh* |  | Cell wall associated fibronectin binding protein |  | N315 |
|  |  | *emp* |  | Secretory extracellular matrix and plasma binding protein |  | COL |
|  |  | *epbS* |  | Elastin-binding protein |  | NCTC 8325 |
|  |  | *fnpA* |  | Fibronectin-binding protein A |  | MW2 |
|  |  | *fnpB* |  | Hypothetical protein |  | N315 |
|  |  | *map* |  | Fibronectin-binding protein |  | COL |
|  |  | *SACOL_0507* |  | LysM domain-containing fibronectin-binding protein |  | COL |
|  |  | *sasA* |  | Serine-rich adhesion for platelets |  | USA300 FPR3757 |
|  |  | *sasC* |  | Cell wall anchor family protein |  | COL |
|  |  | *sasF* |  | Surface protein F |  | MSSA476 |
|  |  | *sasG* |  | Cell wall surface anchor family protein |  | COL |
|  |  | *sdrC* |  | Ser-Asp rich fibrinogen-binding bone sialoprotein-binding protein |  | MW2 |
|  |  | *sdrD* |  | Ser-Asp rich fibrinogen-binding bone sialoprotein-binding protein |  | MW2 |
|  |  | *sdrE* |  | Ser-Asp rich fibrinogen-binding bone sialoprotein-binding protein |  | MW2 |
|  |  | *sraP* |  | LPXTG Cell wall surface anchor family protein |  | COL |
|  |  | *srtA* |  | Sortase A |  | COL |
|  |  | *vWbp* |  | Secreted von Willebrand factor-binding protein precursor |  | Newman |
|  |  |  |  |  |  |  |
| Biofilm |  | *atl* |  | Autolysin |  | USA300 FPR3757 |
|  |  | *hysA* |  | Hyaluronate lyase precursor |  | MW2 |
|  |  | *icaA* |  | N-acetylglucosaminyltrasferase |  | MW2 |
|  |  | *icaB* |  | N-deacetylase |  | MW2 |
|  |  | *icaC* |  | Intercellular adhesion protein C |  | MW2 |
|  |  | *icaD* |  | Intercellular adhesion protein D |  | MW2 |
|  |  | *icaR* |  | *ica* operon transcriptional regulator |  | MW2 |
|  |  |  |  |  |  |  |
| Capsular polysaccharide |  | *capA* |  | Capsular polysaccharide synthesis protein CapA |  | RF122 |
|  |  | *capB* |  | Capsular polysaccharide synthesis protein CapB |  | RF122 |
|  |  | *capC* |  | Capsular polysaccharide synthesis protein CapC |  | RF122 |
|  |  | *capD* |  | Capsular polysaccharide synthesis protein CapD |  | RF122 |
|  |  | *capE* |  | Capsular polysaccharide synthesis protein CapE |  | MRSA252 |
|  |  | *capF* |  | Capsular polysaccharide synthesis protein CapF |  | MRSA252 |
|  |  | *capG* |  | Capsular polysaccharide synthesis protein CapG |  | MRSA252 |
|  |  | *capK* |  | Capsular polysaccharide synthesis protein CapK |  | Newman |
|  |  | *capL* |  | Capsular polysaccharide synthesis protein CapL |  | MRSA252 |
|  |  | *capM* |  | Capsular polysaccharide synthesis protein CapM |  | MRSA252 |
|  |  | *capN* |  | Capsular polysaccharide synthesis protein CapN |  | MRSA252 |
|  |  | *capO* |  | Capsular polysaccharide synthesis protein CapO |  | MRSA252 |
|  |  | *capP* |  | Capsular polysaccharide synthesis protein CapP |  | MRSA252 |
|  |  | *cap5A* |  | Capsular polysaccharide synthesis protein Cap5A |  | USA300 FPR3757 |
|  |  | *cap5B* |  | Capsular polysaccharide synthesis protein Cap5B |  | USA300 FPR3757 |
|  |  | *cap5C* |  | Capsular polysaccharide synthesis protein Cap5C |  | USA300 FPR3757 |
|  |  | *cap5D* |  | Capsular polysaccharide synthesis protein Cap5D |  | NCTC 8325 |
|  |  | *cap5E* |  | Capsular polysaccharide synthesis protein Cap5E |  | COL |
|  |  | *cap5F* |  | Capsular polysaccharide synthesis protein Cap5F |  | USA300 FPR3757 |
|  |  | *cap5G* |  | Capsular polysaccharide synthesis protein Cap5G |  | USA300 FPR3757 |
|  |  | *cap5H* |  | Capsular polysaccharide synthesis protein Cap5H |  | USA300 FPR3757 |
|  |  | *cap5I* |  | Capsular polysaccharide synthesis protein Cap5I |  | USA300 FPR3757 |
|  |  | *cap5J* |  | Capsular polysaccharide synthesis protein Cap5J |  | USA300 FPR3757 |
|  |  | *cap5K* |  | Capsular polysaccharide synthesis protein Cap5K |  | USA300 FPR3757 |
|  |  | *cap5L* |  | Capsular polysaccharide synthesis protein Cap5L |  | USA300 FPR3757 |
|  |  | *cap5M* |  | Capsular polysaccharide synthesis protein Cap5M |  | USA300 FPR3757 |
|  |  | *cap5N* |  | Capsular polysaccharide synthesis protein Cap5N |  | USA300 FPR3757 |
|  |  | *cap5O* |  | Capsular polysaccharide synthesis protein Cap5O |  | USA300 FPR3757 |
|  |  | *cap5P* |  | Capsular polysaccharide synthesis protein Cap5P |  | USA300 FPR3757 |
|  |  | *cap8A* |  | Capsular polysaccharide synthesis protein Cap8A |  | MW2 |
|  |  | *cap8B* |  | Capsular polysaccharide synthesis protein Cap8B |  | MW2 |
|  |  | *cap8C* |  | Capsular polysaccharide synthesis protein Cap8C |  | MW2 |
|  |  | *cap8D* |  | Capsular polysaccharide synthesis protein Cap8D |  | MW2 |
|  |  | *cap8E* |  | Capsular polysaccharide synthesis protein Cap8E |  | MW2 |
|  |  | *cap8F* |  | Capsular polysaccharide synthesis protein Cap8F |  | MW2 |
|  |  | *cap8G* |  | Capsular polysaccharide synthesis protein Cap8G |  | MW2 |
|  |  | *cap8H* |  | Capsular polysaccharide synthesis protein Cap8H copy 1 |  | MW2 |
|  |  | *cap8H* |  | Capsular polysaccharide synthesis protein Cap8H copy 2 |  | MW2 |
|  |  | *cap8I* |  | Capsular polysaccharide synthesis protein Cap8I copy 1 |  | MW2 |
|  |  | *cap8I* |  | Capsular polysaccharide synthesis protein Cap8I copy 2 |  | MW2 |
|  |  | *cap8J* |  | Capsular polysaccharide synthesis protein Cap8J copy 1 |  | MW2 |
|  |  | *cap8J* |  | Capsular polysaccharide synthesis protein Cap8J copy 2 |  | MW2 |
|  |  | *cap8K* |  | Capsular polysaccharide synthesis protein Cap8K copy 1 |  | MW2 |
|  |  | *cap8k* |  | Capsular polysaccharide synthesis protein Cap8K copy 2 |  | MW2 |
|  |  | *cap8L* |  | Capsular polysaccharide synthesis protein Cap8L |  | MW2 |
|  |  | *cap8M* |  | Capsular polysaccharide synthesis protein Cap8M |  | MW2 |
|  |  | *cap8N* |  | Capsular polysaccharide synthesis protein Cap8N |  | MW2 |
|  |  | *cap8O* |  | Capsular polysaccharide synthesis protein Cap8O |  | MW2 |
|  |  | *cap8P* |  | Capsular polysaccharide synthesis protein Cap8P |  | MW2 |
|  |  | *SAOUHSC_00120* |  | UDP-N-acetylglucosamine 2-epimerase |  | NCTC 8325 |
|  |  | *SAURJH9_0143* |  | Hypothetical protein |  | JH9 |
|  |  | *tagO* |  | Undecaprenyl-phosphate N-acetylglucosamynl-1-phosphate transferase |  | COL |
|  |  | *tarK* |  | Ribitol-phosphate polymerase |  | HU-85a |
|  |  |  |  |  |  |  |
| Cytotoxin/hemolysin |  | *ebp* |  | Cell surface elastin binding protein |  | MW2 |
|  |  | *hla/hly* |  | Alpha-hemolysin precursor |  | MW2 |
|  |  | *hlb* |  | Truncated beta-hemolysin |  | USA300 FPR3757 |
|  |  | *hld* |  | Delta-hemolysin |  | MW2 |
|  |  | *hlgA* |  | Gamma-hemolysin chain II precursor |  | MW2 |
|  |  | *hlgB* |  | Gamma-hemolysin component C |  | MW2 |
|  |  | *hlgC* |  | Gamma-hemolysin component B |  | MW2 |
|  |  | *lukA/G* |  | β-channel forming cytolysin component B |  | MI |
|  |  | *lukB/H* |  | β-channel forming cytolysin |  | MI |
|  |  | *lukD* |  | Leukotoxin D |  | USA300 FPR3757 |
|  |  | *lukE* |  | Leukotoxin E |  | USA300 FPR3757 |
|  |  | *lukF-PV* |  | Panton-Valentine leukocidin precursor chain F |  | MW2 |
|  |  | *lukM* |  | Leukocidin chain M precursor |  | RF122 |
|  |  | *lukP* |  | Equid-specific leukocidin P |  | IMT39637 |
|  |  | *lukQ* |  | Equid-specific leukocidin Q |  | IMT39173 |
|  |  | *lukS* |  | Panton-Valentine leukocidin precursor chain S |  | MW2 |
|  |  | *SAUSA300_1974* |  | Leukocidin/hemolysin toxin family protein |  | USA300 FPR3757 |
|  |  |  |  |  |  |  |
| Immune modulator/Superantigen |  | *chp* |  | Chemotaxis-inhibiting protein |  | Newman |
|  |  | *edinB* |  | Epidermal cell differentiation inhibitor B |  | TX018 |
|  |  | *efb* |  | Fibrinogen-binding protein |  | USA300 FPR3757 |
|  |  | *etd* |  | Exfoliative toxin D |  | Y86-2 |
|  |  | *geh* |  | Glycerol-ester hydrolase |  | MW2 |
|  |  | *psm α1* |  | Phenol-soluble modulin α1 peptide |  | JP1 |
|  |  | *psm α2* |  | Phenol-soluble modulin α2 peptide |  | JP1 |
|  |  | *psm α3* |  | Phenol-soluble modulin α3 peptide |  | JP1 |
|  |  | *psm α4* |  | Phenol-soluble modulin α4 peptide |  | JP1 |
|  |  | *psm β* |  | Phenol-soluble modulin β peptide |  | N315 |
|  |  | *psm mec* |  | Phenol-soluble modulin peptide |  | N315 |
|  |  | *sak* |  | Staphylokinase precursor |  | MW2 |
|  |  | *sbi* |  | IgG-binding protein |  | MW2 |
|  |  | *scn* |  | Complement inhibitor SCIN |  | Newman |
|  |  | *sea* |  | *Staphylococcal* enterotoxin A precursor |  | MW2 |
|  |  | *seb* |  | *Staphylococcal* enterotoxin B |  | S6 |
|  |  | *sec* |  | *Staphylococcal* enterotoxin C |  | MW2 |
|  |  | *sed* |  | *Staphylococcal* enterotoxin D |  | RN4220 |
|  |  | *see* |  | *Staphylococcal* enterotoxin E |  | SA-120 |
|  |  | *seg* |  | *Staphylococcal* enterotoxin G |  | RF122 |
|  |  | *seh* |  | *Staphylococcal* enterotoxin H |  | MW2 |
|  |  | *sei* |  | *Staphylococcal* enterotoxin I |  | RF122 |
|  |  | *seJ* |  | *Staphylococcal* enterotoxin J |  | TX123 |
|  |  | *selk* |  | *Staphylococcal* enterotoxin K |  | MW2 |
|  |  | *sell* |  | *Staphylococcal* enterotoxin L |  | MW2 |
|  |  | *selm* |  | *Staphylococcal* enterotoxin M |  | N315 |
|  |  | *seln* |  | *Staphylococcal* enterotoxin N |  | RF122 |
|  |  | *selo* |  | *Staphylococcal* enterotoxin O |  | RF122 |
|  |  | *selp* |  | *Staphylococcal* enterotoxin P |  | N315 |
|  |  | *selq* |  | *Staphylococcal* enterotoxin Q |  | USA300 FPR3757 |
|  |  | *ser* |  | *Staphylococcal* enterotoxin R |  | TX123 |
|  |  | *selt* |  | *Staphylococcal* enterotoxin T |  | AH1 |
|  |  | *seu* |  | *Staphylococcal* enterotoxin U |  | RF122 |
|  |  | *selv* |  | *Staphylococcal* enterotoxin V |  | 363P |
|  |  | *selx* |  | *Staphylococcal* enterotoxin X |  | 363P |
|  |  | *sely* |  | *Staphylococcal* enterotoxin Y |  | 364P |
|  |  | *spa* |  | IgG binding protein A precursor |  | N315 |
|  |  | *ssl3* |  | Superantigen-like protein 3 |  | MW2 |
|  |  | *ssl8* |  | Superantigen-like protein 8 |  | MW2 |
|  |  | *ssl9* |  | Superantigen-like protein 9 |  | MW2 |
|  |  | *tsst-1* |  | Toxic shock syndrome toxin 1 |  | N315 |
|  |  | *ψent1* |  | Enterotoxin *ψent*1 |  | N315 |
|  |  | *ψent2* |  | Enterotoxin *ψent*2 |  | N315 |
|  |  |  |  |  |  |  |
| Iron scavenging/metabolism |  | *arcC* |  | Carbamate kinase 2 |  | RF122 |
|  |  | *arcD* |  | Arginine/ornithine antiporter |  | NCTC 8350 |
|  |  | *fhuD* |  | Iron-regulated surface determinant |  | NCTC 8325 |
|  |  | *htsA* |  | Ferrichrome ABC transporter lipoprotein |  | Newman |
|  |  | *htsB* |  | Ferrichrome ABC transporter permease |  | Newman |
|  |  | *htsC* |  | Ferrichrome ABC transporter permease |  | Newman |
|  |  | *isdA* |  | Iron-regulated surface determinant A |  | Newman |
|  |  | *isdB* |  | Iron-regulated surface determinant B |  | Newman |
|  |  | *isdC* |  | Iron-regulated surface determinant C |  | Newman |
|  |  | *isdD* |  | Iron-regulated surface determinant D |  | MW2 |
|  |  | *isdE* |  | Iron-regulated surface determinant E |  | Newman |
|  |  | *isdF* |  | Iron-regulated surface determinant F |  | MW2 |
|  |  | *isdG* |  | Iron-regulated surface determinant G |  | Newman |
|  |  | *isdH* |  | Heme-degrading monooxygenase |  | Newman |
|  |  | *isdI* |  | Haptoglobin-binding surface anchored protein |  | Newman |
|  |  | *mntC* |  | Mn transport system membrane protein |  | VSRa |
|  |  | *sbnA* |  | O-Acetyl-serine sulfhydrylase |  | Newman |
|  |  | *sbnB* |  | Ornithine cyclodeaminase |  | Newman |
|  |  | *sbnC* |  | Siderophore biosynthesis *IucC* family protein |  | Newman |
|  |  | *sbnD* |  | Membrane transporter protein |  | Newman |
|  |  | *sbnE* |  | Siderophore biosynthesis IucA family protein |  | Newman |
|  |  | *sbnF* |  | Siderophore biosynthesis *IucC* family protein |  | Newman |
|  |  | *sbnG* |  | 2-dehydro-3-deoxyglucarate aldolase |  | Newman |
|  |  | *sbnH* |  | Diaminopimelate decarboxylase |  | Newman |
|  |  | *sfaA* |  | Staphyloferrin A |  | Newman |
|  |  | *sfaB* |  | Staphyloferrin A |  | Newman |
|  |  | *sfaC* |  | Staphyloferrin A |  | Newman |
|  |  | *sfaD* |  | Staphyloferrin A |  | Newman |
|  |  | *sirA* |  | Siderophore ABC transporter binding protein |  | Newman |
|  |  | *sirB* |  | Siderophore ABC transporter permease protein |  | Newman |
|  |  | *sirC* |  | Siderophore ABC transporter permease protein |  | Newman |
|  |  | *srtB* |  | NPQTN specific sortase B |  | MW2 |
|  |  | *sstD* |  | Class III substrate binding protein family |  | MRSA252 |
|  |  |  |  |  |  |  |
| Protease |  | *aur* |  | Zinc metalloproteinase aureolysin |  | MW2 |
|  |  | *eta* |  | Exfoliative toxin A |  | USA300 FPR3757 |
|  |  | *splA* |  | Serine protease SplA |  | USA300 FPR3757 |
|  |  | *splB* |  | Serine protease SplB |  | USA300 FPR3757 |
|  |  | *splC* |  | Serine protease SplC |  | USA300 FPR3757 |
|  |  | *splD* |  | Serine protease SplD |  | USA300 FPR3757 |
|  |  | *splE* |  | Serine protease SplE |  | USA300 FPR3757 |
|  |  | *splF* |  | Serine protease SplF |  | USA300 FPR3757 |
|  |  | *sspA* |  | Glutamyl endopeptidase V8 |  | MW2 |
|  |  | *sspB* |  | Staphopain cysteine proteinase |  | MW2 |
|  |  |  |  |  |  |  |
| Protease Inhibitor |  | *sspC* |  | Staphostatin B |  | MW2 |
|  |  |  |  |  |  |  |
| Toxin |  | *adsA* |  | Adenosine synthase A |  | MW2 |
|  |  | *lip* |  | Triacylglycerol lipase precursor |  | MW2 |
|  |  | *nuc* |  | Thermonuclease precursor |  | USA300 FPR3757 |
|  |  | *set1* |  | *Staphylococcal* exotoxin 1 |  | MRSA252 |
|  |  | *set2* |  | *Staphylococcal* exotoxin 2 |  | MRSA252 |
|  |  | *set3* |  | *Staphylococcal* exotoxin 3 |  | MRSA252 |
|  |  | *set4* |  | *Staphylococcal* exotoxin 4 |  | MRSA252 |
|  |  | *set5* |  | *Staphylococcal* exotoxin 5 |  | MRSA252 |
|  |  | *set6* |  | *Staphylococcal* exotoxin 6 |  | N315 |
|  |  | *set7* |  | *Staphylococcal* exotoxin 7 |  | N315 |
|  |  | *set8* |  | *Staphylococcal* exotoxin 8 |  | NCTC 8325 |
|  |  | *set9* |  | *Staphylococcal* exotoxin 9 |  | N315 |
|  |  | *set10* |  | *Staphylococcal* exotoxin 10 |  | N315 |
|  |  | *set11* |  | *Staphylococcal* exotoxin 11 |  | N315 |
|  |  | *set12* |  | *Staphylococcal* exotoxin 12 |  | N315 |
|  |  | *set13* |  | *Staphylococcal* exotoxin 13 |  | NCTC 8325 |
|  |  | *set14* |  | *Staphylococcal* exotoxin 14 |  | N315 |
|  |  | *set15* |  | *Staphylococcal* exotoxin 15 |  | N315 |
|  |  | *set16* |  | *Staphylococcal* exotoxin 11 |  | RF122 |
|  |  | *set17* |  | *Staphylococcal* exotoxin 10 |  | RF122 |
|  |  | *set18* |  | *Staphylococcal* exotoxin 9 |  | RF122 |
|  |  | *set19* |  | Exotoxin |  | MSSA476 |
|  |  | *set20* |  | *Staphylococcal* exotoxin 7 |  | RF122 |
|  |  | *set21* |  | Exotoxin |  | MSSA476 |
|  |  | *set22* |  | *Staphylococcal* exotoxin 5 |  | RF122 |
|  |  | *set23* |  | *Staphylococcal* exotoxin 4 |  | RF122 |
|  |  | *set24* |  | *Staphylococcal* exotoxin 3 |  | RF122 |
|  |  | *set25* |  | *Staphylococcal* exotoxin 2 |  | RF122 |
|  |  | *set26* |  | *Staphylococcal* exotoxin 1 |  | RF122 |
|  |  | *set30* |  | Exotoxin |  | USA300 FPR3757 |
|  |  | *set31* |  | *Staphylococcal* exotoxin 7 |  | USA300 FPR3757 |
|  |  | *set32* |  | Exotoxin |  | USA300 FPR3757 |
|  |  | *set33* |  | Exotoxin |  | USA300 FPR3757 |
|  |  | *set34* |  | Exotoxin |  | USA300 FPR3757 |
|  |  | *set35* |  | Exotoxin |  | USA300 FPR3757 |
|  |  | *set36* |  | Exotoxin |  | USA300 FPR3757 |
|  |  | *set37* |  | Exotoxin |  | USA300 FPR3757 |
|  |  | *set38* |  | Exotoxin |  | COL |
|  |  | *set39* |  | Exotoxin |  | USA300 FPR3757 |
|  |  | *set40* |  | Exotoxin |  | USA300 FPR3757 |
|  |  |  |  |  |  |  |
| Type VII/ESS secretion system |  | *esaA* |  | Type VII secretion system protein EsaA |  | MW2 |
|  |  | *esaB* |  | Type VII secretion system protein EsaB |  | MW2 |
|  |  | *esaC* |  | Type VII secretion system secreted protein EsaA |  | MW2 |
|  |  | *essA* |  | Monotopic membrane protein EssA |  | MW2 |
|  |  | *essB* |  | Monotopic membrane protein EssB |  | MW2 |
|  |  | *essC* |  | FtsK/SpoIIIE family ATPase |  | MW2 |
|  |  | *essD* |  | Type VII secretion system protein EssD |  | MW2 |
|  |  | *esxA* |  | Type VII secretion system protein EsxA |  | MW2 |
|  |  | *esxB* |  | Type VII secretion system protein EsxB |  | MW2 |
