# Supplementary material for: Genomics of Staphylococcus aureus ocular isolates
Source: PLoS One. 2021 May 3;16(5):e0250975. doi: 10.1371/journal.pone.0250975 (PMC8092774; doi:10.1371/journal.pone.0250975)
Supplement: S4 Table — (DOCX) [file pone.0250975.s008.docx]

**S4 Table**. Virulence factors associated with one or more strain groups (SGs).

| ***Gene*** | ***Function*** | ***Prevalence SG1*** | ***Prevalence SG2*** | ***Prevalence SG3*** | ***Prevalence SG4*** | ***Adj p-value (BH)*** |
| --- | --- | --- | --- | --- | --- | --- |
| *ebp* | Adherence | 1.000 | 1.000 | 1.000 | 0.875 | 9.66E-06 |
| *sdrD* | Adherence | 0.972 | 0.960 | 0.955 | 0.781 | 4.32E-04 |
| *sdrE* | Adherence | 0.972 | 0.933 | 0.985 | 0.859 | 2.50E-02 |
| *sea* | Immune modulator/superantigen | 0.310 | 0.120 | 0.254 | 0.172 | 3.67E-02 |
| *sak* | Immune modulator | 0.859 | 0.947 | 0.597 | 0.813 | 4.05E-06 |
| *seb* | Immune modulator/superantigen | 0.254 | 0.053 | 0.269 | 0.172 | 1.82E-03 |
| *tsst-1* | Immune modulator/super antigen | 0.141 | 0.013 | 0.060 | 0.234 | 1.46E-04 |
| *esaC* | type VII/ESS secretion system | 0.986 | 0.987 | 0.313 | 0.422 | 1.17E-31 |
| *esxB* | type VII/ESS secretion system | 0.986 | 0.987 | 0.313 | 0.422 | 1.17E-31 |
| *vWbp* | Adherence | 0.944 | 0.907 | 0.522 | 0.734 | 1.74E-09 |
| *sell* | Immune modulator/superantigen | 0.225 | 0.000 | 0.075 | 0.125 | 3.19E-05 |
| *sec* | Immune modulator/superantigen | 0.254 | 0.053 | 0.269 | 0.172 | 1.82E-03 |
| *fnbB* | Adherence | 0.915 | 1.000 | 0.851 | 0.563 | 7.13E-12 |
| *coa* | Adherence | 0.958 | 0.987 | 0.358 | 0.203 | 1.88E-36 |
| *splF* | Protease | 0.986 | 0.987 | 0.731 | 0.438 | 1.21E-19 |
| *splE* | Protease | 0.986 | 0.987 | 0.731 | 0.438 | 1.21E-19 |
| *splD* | Protease | 0.986 | 0.987 | 0.731 | 0.438 | 1.21E-19 |
| *splC* | Protease | 0.986 | 0.987 | 0.731 | 0.438 | 1.21E-19 |
| *splB* | Protease | 0.986 | 0.987 | 0.746 | 0.125 | 5.18E-38 |
| *splA* | Protease | 0.986 | 0.987 | 0.731 | 0.125 | 5.26E-38 |
| *lukD* | Cytotoxin/hemolysin | 1.000 | 1.000 | 0.836 | 0.953 | 7.85E-06 |
| *set40* | Toxin | 0.986 | 0.920 | 0.284 | 0.406 | 1.82E-28 |
| *set38* | Toxin | 1.000 | 0.987 | 0.970 | 0.844 | 9.32E-05 |
| *set37* | Toxin | 1.000 | 0.987 | 0.925 | 0.422 | 5.45E-23 |
| *set36* | Toxin | 1.000 | 0.987 | 1.000 | 0.922 | 5.19E-03 |
| *set32* | Toxin | 1.000 | 1.000 | 0.910 | 0.219 | 1.60E-36 |
| *set26* | Toxin | 0.986 | 0.080 | 0.731 | 0.578 | 2.53E-33 |
| *set23* | Toxin | 1.000 | 0.987 | 0.925 | 0.422 | 5.45E-23 |
| *ssl8* | Immune modulator/superantigen | 1.000 | 1.000 | 0.955 | 0.359 | 3.19E-29 |
| *set22* | Toxin | 1.000 | 0.987 | 1.000 | 0.922 | 5.19E-03 |
| *set18* | Toxin | 1.000 | 1.000 | 0.925 | 0.219 | 2.56E-37 |
| *ssl3* | Immune modulator/superantigen | 1.000 | 1.000 | 0.910 | 0.219 | 1.60E-36 |
| *set15* | Toxin | 1.000 | 0.920 | 0.448 | 0.234 | 4.83E-31 |
| *set12* | Toxin | 1.000 | 0.987 | 0.925 | 0.422 | 5.45E-23 |
| *set11* | Toxin | 1.000 | 0.987 | 1.000 | 0.922 | 5.19E-03 |
| *set8* | Toxin | 1.000 | 1.000 | 0.910 | 0.219 | 1.60E-36 |
| *set1* | Toxin | 1.000 | 0.987 | 1.000 | 0.922 | 5.19E-03 |
| *Ψent2* | Immune modulator/superantigen | 1.000 | 0.000 | 0.015 | 0.859 | 4.41E-68 |
| *Ψent1* | Immune modulator/superantigen | 1.000 | 0.000 | 0.000 | 0.859 | 4.95E-70 |
| *selp* | Immune modulator/superantigen | 0.239 | 0.040 | 0.104 | 0.000 | 7.85E-06 |
| *selo* | Immune modulator/superantigen | 1.000 | 0.000 | 0.030 | 0.859 | 1.72E-66 |
| *seln* | Immune modulator/superantigen | 1.000 | 0.000 | 0.000 | 0.859 | 4.95E-70 |
| *selm* | Immune modulator/superantigen | 1.000 | 0.000 | 0.015 | 0.859 | 4.41E-68 |
| *sei* | Immune modulator/superantigen | 1.000 | 0.000 | 0.015 | 0.859 | 4.41E-68 |
| *seg* | Immune modulator/superantigen | 1.000 | 0.000 | 0.000 | 0.813 | 9.74E-68 |
| *cap5H* | Capsular polysaccharide | 1.000 | 1.000 | 0.015 | 0.313 | 3.40E-61 |
| *cap5I* | Capsular polysaccharide | 1.000 | 1.000 | 0.015 | 0.313 | 3.40E-61 |
| *cap5J* | Capsular polysaccharide | 1.000 | 1.000 | 0.015 | 0.313 | 3.40E-61 |
| *cap5K* | Capsular polysaccharide | 1.000 | 1.000 | 0.015 | 0.313 | 3.40E-61 |
| *SaurJH9* | capsular biosynthesis protein | 1.000 | 1.000 | 0.015 | 0.313 | 3.40E-61 |
| *capK* | Capsular polysaccharide | 1.000 | 1.000 | 0.015 | 0.313 | 3.40E-61 |
| *sasG* | Adherence | 0.620 | 0.573 | 0.313 | 0.000 | 2.52E-18 |
| *sraP* | Adherence | 0.930 | 0.973 | 0.910 | 0.578 | 4.90E-10 |
| *selv* | Immune modulator/superantigen | 1.000 | 0.000 | 0.015 | 0.859 | 4.41E-68 |
| *epbS* | Adherence | 1.000 | 1.000 | 1.000 | 0.875 | 9.66E-06 |
| *sasA* | Adherence | 0.972 | 0.880 | 0.910 | 0.578 | 7.46E-09 |
| *essD* | Toxin | 0.986 | 0.987 | 0.313 | 0.422 | 1.17E-31 |
| *selX* | Immune modulator/superantigen | 1.000 | 1.000 | 0.970 | 0.547 | 1.40E-19 |
| *seu* | Immune modulator/superantigen | 1.000 | 0.000 | 0.000 | 0.859 | 4.95E-70 |
| *lukQ* | Cytotoxin | 1.000 | 1.000 | 0.836 | 0.953 | 7.85E-06 |
| *psm* | Toxin | 0.408 | 0.000 | 0.179 | 0.016 | 7.97E-14 |
| *set35* | Toxin | 0.000 | 0.880 | 0.627 | 0.141 | 5.55E-38 |
| *set21* | Toxin | 0.000 | 0.880 | 0.627 | 0.141 | 5.55E-38 |
| *selk* | Immune modulator/superantigen | 0.000 | 0.680 | 0.254 | 0.000 | 7.27E-29 |
| *selq* | Immune modulator/superantigen | 0.000 | 0.680 | 0.254 | 0.000 | 7.27E-29 |
| *cna* | Adherence | 0.014 | 0.000 | 0.343 | 0.813 | 3.18E-35 |
| *cap8H* | capsular polysaccharide | 0.000 | 0.000 | 0.970 | 0.688 | 5.85E-59 |
| *cap8I* | capsular polysaccharide | 0.000 | 0.000 | 0.985 | 0.688 | 3.40E-61 |
| *cap8J* | capsular polysaccharide | 0.000 | 0.000 | 0.985 | 0.688 | 3.40E-61 |
| *cap8K* | capsular polysaccharide | 0.000 | 0.000 | 0.985 | 0.688 | 3.40E-61 |
| *selY* | Immune modulator/superantigen | 0.000 | 0.000 | 0.239 | 0.172 | 4.07E-09 |
| *she* | Immune modulator/superantigen | 0.000 | 0.000 | 0.090 | 0.016 | 1.82E-03 |
| *sed* | Immune modulator/superantigen | 0.225 | 0.053 | 0.000 | 0.000 | 1.05E-07 |
| *seJ* | Immune modulator/superantigen | 0.239 | 0.053 | 0.000 | 0.000 | 2.74E-08 |
| *ser* | Immune modulator/superantigen | 0.239 | 0.053 | 0.015 | 0.000 | 3.30E-07 |
